# Insights into Single-Electron-Transfer Processes in Frustrated Lewis Pair Chemistry and Related Donor–Acceptor Systems in Main Group Chemistry

**DOI:** 10.1021/acs.chemrev.3c00217

**Published:** 2023-07-11

**Authors:** Lars J. C. van der Zee, Sanjukta Pahar, Emma Richards, Rebecca L. Melen, J. Chris Slootweg

**Affiliations:** † Van ’t Hoff Institute for Molecular Sciences, University of Amsterdam, P.O. Box 94157, 1090 GD Amsterdam, The Netherlands; ‡ Cardiff Catalysis Institute, Cardiff University, Translational Research Hub, Maindy Road, Cathays, Cardiff, CF24 4HQ Wales, United Kingdom

## Abstract

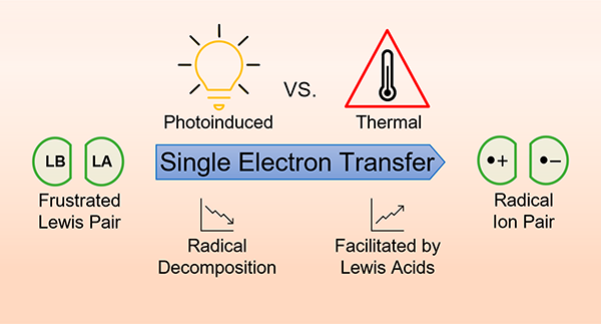

The activation and utilization of substrates mediated by Frustrated Lewis Pairs (FLPs) was initially believed to occur solely via a two-electron, cooperative mechanism. More recently, the occurrence of a single-electron transfer (SET) from the Lewis base to the Lewis acid was observed, indicating that mechanisms that proceed via one-electron-transfer processes are also feasible. As such, SET in FLP systems leads to the formation of radical ion pairs, which have recently been more frequently observed. In this review, we aim to discuss the seminal findings regarding the recently established insights into the SET processes in FLP chemistry as well as highlight examples of this radical formation process. In addition, applications of reported main group radicals will also be reviewed and discussed in the context of the understanding of SET processes in FLP systems.

## 1 Introduction

**Scheme 1:**
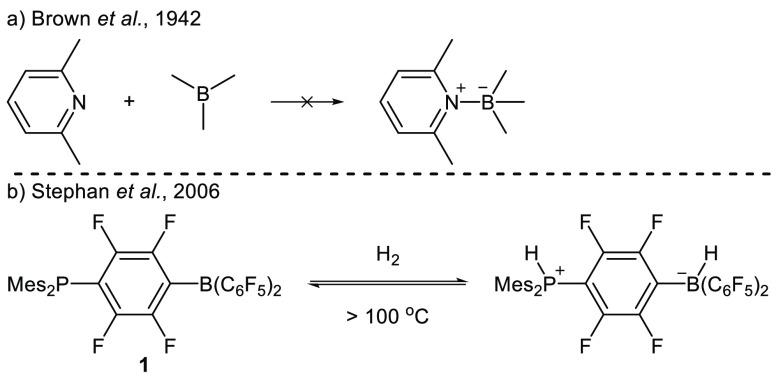
First Report on (A) Prevention of Adduct Formation and (B) Reactivity Induced by a Sterically Encumbered Lewis Acid and Base

In 1942, Brown and co-workers were the first to observe the influence of steric hindrance on the interaction between Lewis acids and Lewis bases.^1^ They showed that the combination of trimethyl borane and lutidine are unable to form a classic Lewis acid–base adduct, even at temperatures as low as −80 °C (Scheme 1A). The authors explained these findings by suggesting that steric hindrance prevents the formation of the Lewis adduct. Decades later, in 2006, the group of Stephan et al. showed for the first time that a combination of sterically encumbered Lewis acids and bases can also induce intriguing reactivity.^2^ They demonstrated that phosphine borane **1** can bind dihydrogen and release it again upon heating (Scheme 1B). A year later, Stephan et al. introduced the term *Frustrated Lewis Pairs (FLPs)* to highlight cases in which the Lewis acid and base are unable to form a classical Lewis adduct.^3^ Since these seminal reports, the use of FLPs has been widely explored and rejuvenated the field of main group chemistry and catalysis.^4−7^

**Scheme 2:**
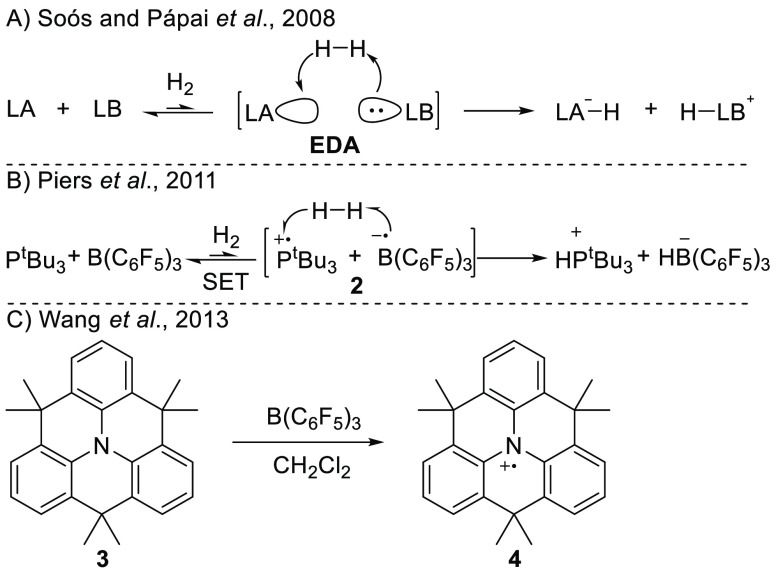
(A) Two-Electron Reactivity of an FLP System via the Formation of an Encounter Complex, **EDA**; (B) First Postulation of Radical Formation in an FLP System; (C) Single-Electron Oxidation of **3** by B(C_6_F_5_)_3_ Leads to the Formation of Radical Cation **4**

Typically, the reactivity of FLP systems is described by two-electron chemistry, where the Lewis acid and base act cooperatively.^8,9^ In this mechanism, it is now recognized that the Lewis acid and base first form a so-called encounter complex **EDA**, as shown in Scheme 2A.^10,11^ Next, the substrate is heterolytically activated by simultaneous donation of electron density from the Lewis base lone pair into an antibonding orbital of the substate (e.g., the H–H σ*-orbital of H_2_) and the acceptance of electron density from a bonding orbital of the substrate (e.g., the H–H σ-orbital of H_2_) by the Lewis acid. In addition to these early reports, Piers et al. postulated in 2011 that FLPs can also react via one-electron pathways.^12^ For the activation of dihydrogen by the P^t^Bu_3_/B(C_6_F_5_)_3_ FLP, the authors envisioned that one-electron oxidation of the phosphine by the borane via a single-electron transfer (SET) event occurs as the first reaction step, yielding the radical ion pair P^t^Bu_3_^•+^/B(C_6_F_5_)_3_^•–^**2** (Scheme 2B). Subsequently, dihydrogen is activated homolytically to yield the same phosphonium borohydride salt [^t^Bu_3_PH][HB(C_6_F_5_)_3_] as obtained via the heterolytic cleavage of the H–H bond. Piers et al. presumed that reaction progress through this radical mechanism was unlikely to be the major contribution due to the large disparity in redox potentials between the P^t^Bu_3_ (0.90 V vs Fc/Fc^+^ in MeCN^13^) and B(C_6_F_5_)_3_ (−1.79 V vs Fc/Fc^+^ in DCM^14^). This difference results in only a small amount of the intermediate radical ion pair **2** being present in solution, which does not correlate with the observation that P^t^Bu_3_/B(C_6_F_5_)_3_ promptly reacts with dihydrogen.^15^ Later, Slootweg and co-workers showed with ultrafast transient absorption spectroscopy that the lifetime of the radical ion pair P^t^Bu_3_^•+^/B(C_6_F_5_)_3_^•–^**2** is very short (6 ps) due to rapid back electron transfer to the ground-state electron donor–acceptor (**EDA**) complex [P^t^Bu_3_, B(C_6_F_5_)_3_], which prevents subsequent radical reactivity.^16^ Therefore, in this case, dihydrogen is solely activated heterolytically.^17^

**Figure 1:**
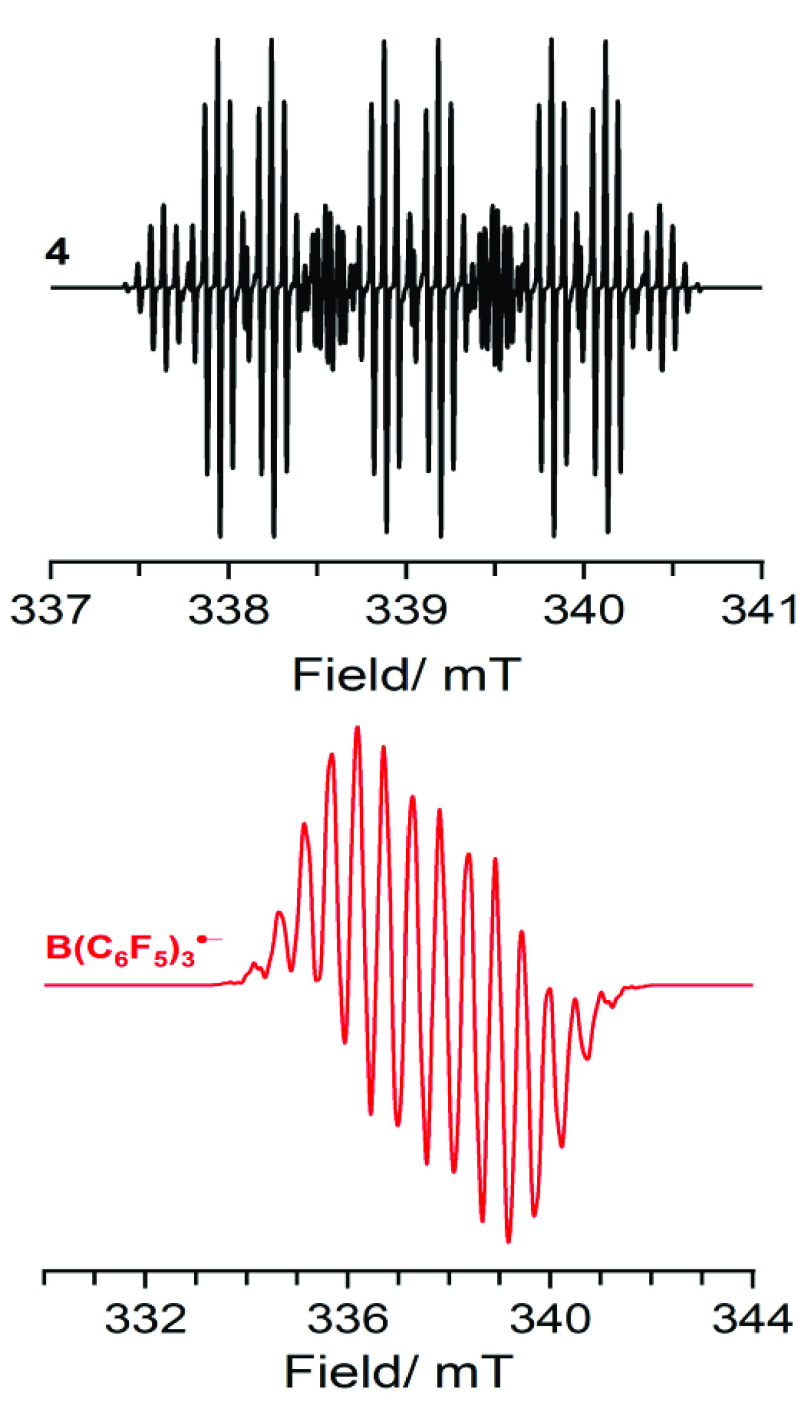
Isotropic CW X-band EPR spectra of radicals **4** and B(C_6_F_5_)_3_^•–^; simulated using data reported in Table 1.

Two years after the proposal of radical ion pairs in FLP chemistry by Piers et al., the Wang group provided the first direct experimental evidence of SET between a Lewis base and Lewis acid.^18^ Namely, upon mixing arylamine **3** and B(C_6_F_5_)_3_ and stirring for 3 days at room temperature, the authors obtained a deep blue solution due to the formation of the amine radical cation **4**, which they proved using electron paramagnetic resonance (EPR) spectroscopy (Scheme 2C; Figure 1; see Table 1 for parameters). The corresponding borane radical anion B(C_6_F_5_)_3_^•–^ was however not observed, which is associated with its facile decomposition. Yet, the EPR spectrum of the B(C_6_F_5_)_3_^•–^ radical anion has been previously reported at low temperatures and is characterized by a rich hyperfine structure originating from the nuclear spin-active ^10,11^B isotopes and fluorine substituents at all positions on the phenyl ring (Figure 1; see Table 1 for parameters).^14,19−21^

**Table 1:** Spin Hamiltonian Parameters for Radical Species Generated during FLP Reactions

radical	*g*_i__s__o_	*a*_iso_/mT^a^	reference
**Phosphorus**			
PMes_3_^•+^	2.009; *g*_||_ = 2.010; *g*_⊥_ = 2.013	23.8; *A*_||_ = 40.30; *A*_⊥_ = 15.98	(33)
P^t^Bu_3_^•+^	2.005; *g*_||_ = 2.0012; *g*_⊥_ = 2.0065	30.00; *A*_||_ = 48.65; *A*_⊥_ = 20.67	(16)
PTipp^•+^	2.007; *g*_||_ = 2.002; *g*_⊥_ = 2.009	22.55; *A*_||_ = 41.58; *A*_⊥_ = 13.03	(20)
(PEt_3_)_2_^•+^	2.008; *g*_||_ = 2.00; *g*_⊥_ = 2.012	45.44; *A*_||_ = 53.76; *A*_⊥_ = 41.27	(83)
(P^t^Bu_3_)_2_^•+^	2.008; *g*_||_ = 2.00; *g*_⊥_ = 2.012	46.18; *A*_||_ = 54.80; *A*_⊥_ = 41.88	(83)
(PMes_3_)_2_^•+^	2.008; *g*_||_ = 2.003; *g*_⊥_ = 2.011	16.54; *A*_||_ = 26.90; *A*_⊥_ = 11.39	(57)
[(*p*-ClC_6_H_4_)N_2_(P^t^Bu_3_)]^•^**(37)**	2.005	P = 0.88; N_1_ = 0.42; N_2_ = 0.36; H_*o*_ = 0.36; Cl = 0.28	(55)
**Nitrogen**			
(C(CH_3_)_2_C_6_H_3_)_3_N **(4)**	2.002	^14^N: 0.94; ^1^H_*p(3)*_: 0.304; ^1^H_*m(6)*_: 0.071	(18)
^t^Bu_3_P–NO^•^	2.0071	^14^N = 1.05; ^31^P = 1.21	(13)
C_6_H_4_(NMe_2_)_2_**(70**^**•+**^**)**	2.0034	^14^N_*(2)*_ = 0.7; ^1^H_*(4)*_ = 0.195; ^1^H_*(12)*_ = 0.655	(75)
(Me_2_PhN–C_6_H_5_)_2_ **(72)**	2.0033	^14^N_*(2)*_ = 0.45	(75)
Br–C_6_H_4_–N(Me)–CH_2_–TMS **(85)**	2.0033	^14^N_*(1)*_ = 0.824; ^1^H_*(3)*_ = 0.738 ;^1^H_*(2)*_ = 0.992; ^1^H_*(2)*_ = 0.345; ^1^H_*(2)*_ = 0.133; Si_*(1)*_ = 0.313	(81)
Br–C_6_H_4_–N(Me)_2_**(88)**	2.0029	^14^N_*(1)*_ = 1.174; ^1^H_*(3)*_ = 0.773; ^1^H_*(2)*_ = 0.414; ^1^H_*(2)*_ = 0.205	(81)
**Boron**			
B(C_6_F_5_)_3_^•–^	2.011	B: 1.10; F_*o(6)*_: 0.46; F_*m(6)*_: 0.13; F_*p(3)*_: 0.53	(19)
**Germanium**			
[BCHGe]^•+^**(47′)**	1.988	^177,179^Hf: 8.5	(59)
**Carbon**			
CPh_3_^•^	1.999	H_*o(6)*_: 0.26; H_*m(6)*_: 0.11; H_*p(3)*_: 0.28	(20)
C(C_6_F_5_)_3_^•^	2.003	n.r.	(52)
C(C_6_H_3_O)_3_^•^**(27)**	2.005	H_*m(6)*_ = 0.091; H_*p(3)*_ = 0.328	(53)
(C_6_H_4_)_2_CH_2_^•^**(42)**	2.006	H_1_ = 2.28; H_3*(2)*_ = 0.33; H_6*(2)*_ = 0.27	(56)
(B(C_6_F_5_)_3_)_2_-9,10-anthraquinone **(70**^**•–**^**)**	2.004	n.r.	(75)

a *a*_iso_/MHz = 10^–9^ (gμ_B_/h) *a*_iso_/mT.

**Scheme 3:**
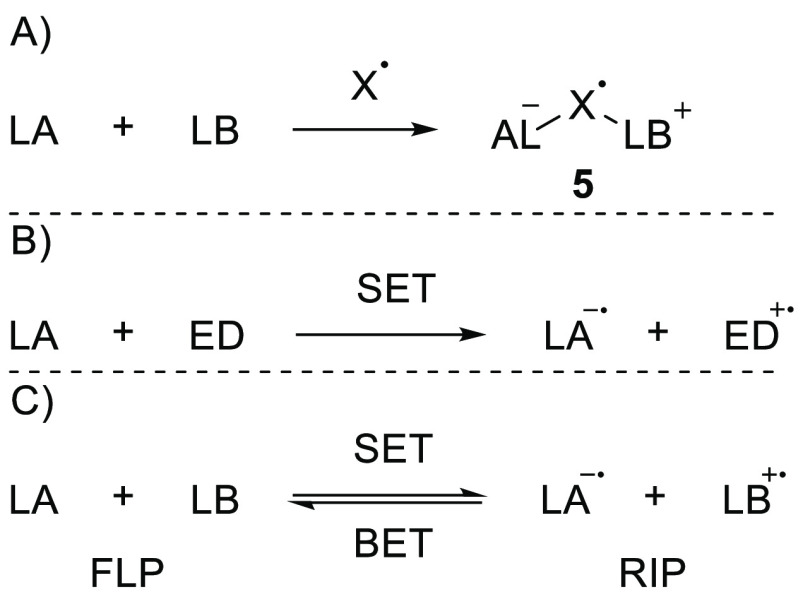
Three Methods to Obtain Radicals in FLP Chemistry: (A) Incorporation of a Radical Substrate into an FLP to Form a Radical “Trimer”; (B) One-Electron Reduction of a Lewis Acid by a Transition-Metal-Based Single-Electron Donor; (C) SET between a LB and LA to Form an RIP LA = Lewis Acid, LB = Lewis base, ED = electron donor, X = radical substrate.

To date, three different approaches for the formation of radicals in FLP chemistry have been identified.^22−25^ First, the FLP can react with a radical substrate X, such as nitric oxide, to yield a “trimer” radical of the form LA–X–LB **5** (Scheme 3A).^26−31^ The second route involves the use of a transition-metal-based electron donor, such as ferrocene or cobaltocene, to reduce the Lewis acid to the corresponding radical anion (Scheme 3B).^23^ Finally, the generation of radical ion pairs (RIPs) can proceed via direct SET between a Lewis base and Lewis acid that function as an electron donor and electron acceptor, respectively (Scheme 3C). These RIPs were coined Frustrated Radical Pairs (FRPs) by Stephan et al. Notably, the radical ion pair can be converted back to the closed-shell FLP system via back electron transfer (BET),^32^ depending on the relative energies of the two states. In this review, to support discussion of the three alternative approaches to radical formation in FLP chemistry, we shall highlight selected main-group chemistry examples where we believe SET mechanisms are prevalent and focus on the mechanistic details of the SET process and the corresponding spectroscopic findings.

## 2 Photoinduced Single-Electron Transfer

**Figure 2:**
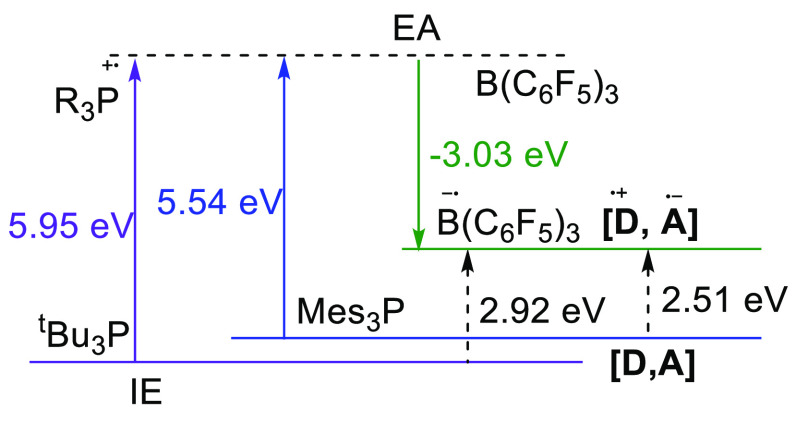
Energy required for the formation of a radical ion pair can be estimated from the electron donor’s ionization energy (IE) and the electron acceptor’s electron affinity (EA).^16^ Calculations at the SCRF/ωB97X-D/6-311+G(d,p) (solvent = toluene) level of theory.

In 2020, Slootweg et al. reported on key insights into the mechanism of SET in FLP chemistry.^16^ Prior to this contribution, Stephan et al. had observed only the phosphine radical cation for the PMes_3_/B(C_6_F_5_)_3_ combination in chlorobenzene.^33^ In-line with the findings of Piers et al., Slootweg et al. concluded that thermally activated SET for this combination of Lewis acid and base is not feasible. Namely, DFT calculations at the SCRF (toluene)/ωB97X-D/6-311+G(d,p) level of theory showed that the electron affinity of B(C_6_F_5_)_3_ is 3.03 eV (conversion factor: 1.00 eV = 23.0621 kcal/mol), while the ionization energy of PMes_3_ is 5.54 eV, resulting in an energy difference between the closed shell PMes_3_/B(C_6_F_5_)_3_ and the radical ion pair PMes_3_^•+^/B(C_6_F_5_)_3_^•–^ of 2.51 eV (57.8 kcal/mol; Figure 2). A similar result was computed for P^t^Bu_3_/B(C_6_F_5_)_3_, for which an even larger energy difference of 2.92 eV (67.2 kcal/mol) for the P^t^Bu_3_^•+^/B(C_6_F_5_)_3_^•–^ RIP was found due to the reduced stabilization of the phosphorus radical cation.^20,21,32^ These energy differences indicate that whereas SET is unlikely to be induced by a thermal reaction, interaction with visible light (λ = 400–800 nm, Δ*E* = 71.4–35.7 kcal/mol) is however feasible.

Inspired by the pioneering work of Mulliken and Kochi et al.,^34,35^ Slootweg et al. realized that the Mulliken theory can be used to explain the observation of these radical ion pairs.^16^ Namely, an electron donor and an electron acceptor (classified as Lewis base and Lewis acid in Frustrated Lewis Pair chemistry) can combine to form an electron donor–acceptor (EDA) complex, which is visualized in Scheme 2A as the encounter complex **EDA**.^34,35^ This EDA complex can be characterized by UV–vis spectroscopy^36^ and shows a new absorption band at longer wavelengths compared to the absorptions of the individual FLP components. Irradiation of the FLP system using selective wavelengths that align with the absorption band of the EDA leads to a photoinduced SET from the electron donor to the acceptor.

**Figure 3:**
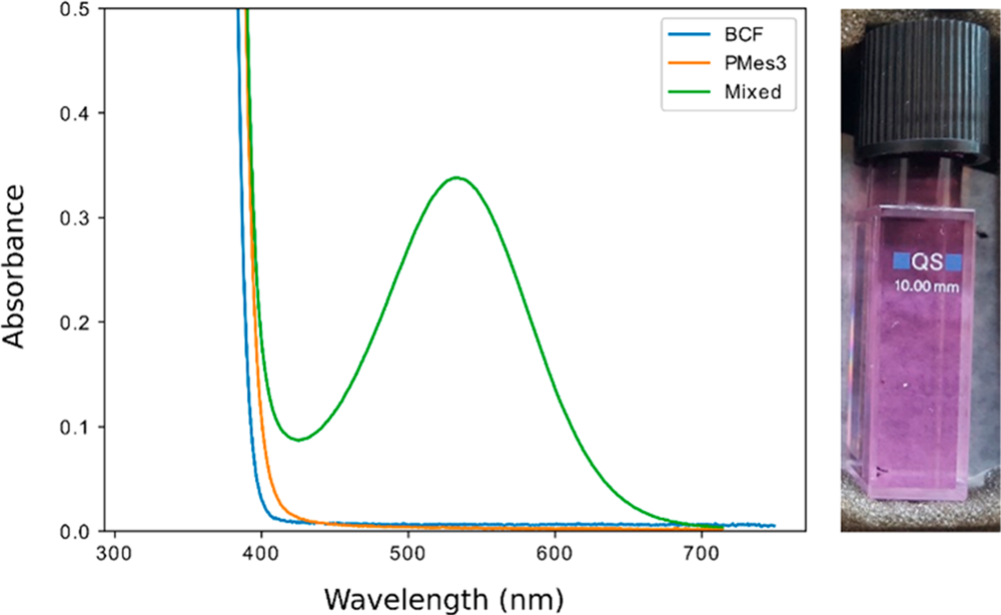
UV–vis absorption spectrum of a 1.5 × 10^–2^ M mixture of B(C_6_F_5_)_3_ and PMes_3_ in toluene, indicating the formation of a new EDA absorption band.

The reported UV–vis spectra of the PMes_3_/B(C_6_F_5_)_3_ (violet) and P^t^Bu_3_/B(C_6_F_5_)_3_ (pale yellow) FLPs in toluene showed a new absorption band to be present for both (at λ_max_ = 534 nm and λ_max_ ≈ 400 nm, respectively; see Figure 3).^16^ Time-dependent DFT (TD-DFT) calculations also predicted the presence of these new absorption bands [PMes_3_/B(C_6_F_5_)_3_: 439 nm (*f*_osc_ = 0.0184); and P^t^Bu_3_/B(C_6_F_5_)_3_: 400 nm (*f*_osc_ = 0.0719)]. Analysis of the frontier molecular orbitals showed that the donor orbital (HOMO) contains the phosphine lone pair, while the acceptor is the empty orbital (LUMO) located on B(C_6_F_5_)_3_, underlining that FLP systems are ideally suited for single-electron-transfer processes.

**Scheme 4:**
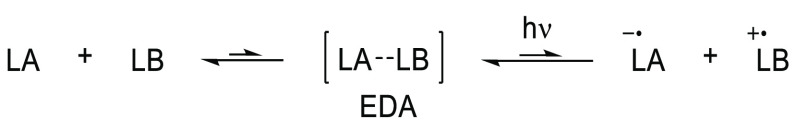
Photolytic Single-Electron Transfer between a Lewis Acid (LA) and a Lewis Base (LB) Following Mulliken Theory As Postulated by Slootweg et al.^16^

**Figure 4:**
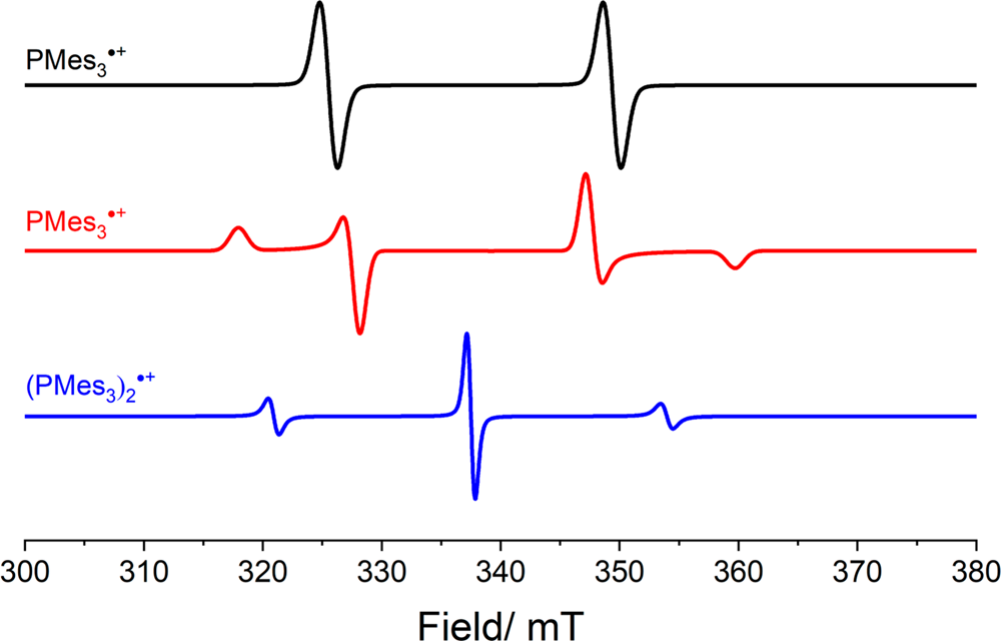
Isotropic (top) and anisotropic (middle) CW X-band EPR spectra of PMes_3_^•+^, and (PMes_3_)_2_^•+^ (bottom); simulated using data reported in Table 1.

To confirm that the SET in these phosphine/borane systems is photoinduced, EPR spectroscopy was performed in toluene at 30 K.^16^ When measuring the samples in the dark, no signals were observed in the EPR spectra, highlighting that the color of the solution stems from the closed-shell EDA complex and not from the presence of radicals. On the other hand, upon irradiation with visible light (390–500 nm), SET was promoted to form the corresponding RIP, as evidenced by the observation of two separate signals in the EPR spectra (Figure 4), which could be assigned for the first time to both the phosphine radical cation and the borane radical anion. Subsequently, by using transient absorption spectroscopy, the lifetime of the radical ion pair state in toluene at room temperature could be determined to be 237 and 6 ps for PMes_3_/B(C_6_F_5_)_3_ and P^t^Bu_3_/B(C_6_F_5_)_3_, respectively. These results indicate that BET occurs quickly after SET, leading to an equilibrium between the ground state and the radical state, which lies heavily on the side of the EDA complex, as shown in Scheme 4. These results prove that the color of these solutions (in the dark) is due to the existence of a charge-transfer band, in contrast to earlier reports where typically the color was assigned to the presence of radicals.^33^

Similarly, an equimolar mixture of Mes_3_P and Al(C_6_F_5_)_3_ in dry toluene or chlorobenzene was reported by Stephan et al. to yield, without exclusion of light, a distinctly purple-colored solution.^33^ The room-temperature EPR spectrum of the reaction mixture showed a doublet resonance with a hyperfine coupling constant of 23.8 mT centered at *g*_iso_ = 2.0089, assigned to PMes_3_^•+^ of the corresponding radical ion pair PMes_3_^•+^/Al(C_6_F_5_)_3_^•–^ (Figure 4).^17^ No evidence of the radical anion Al(C_6_F_5_)_3_^•–^ could be observed via EPR, analogous to the boron-based radical anion.^33^ Based on the findings of the photoinduced SET, we expect the formation of PMes_3_^•+^ in this case is due to irradiation of the charge-transfer band by visible light and that the charge-transfer band causes the purple color. Similar to B(C_6_F_5_)_3_^•–^, the absence of Al(C_6_F_5_)_3_^•–^ can be explained by facile decomposition.

Subsequently, Marques and Ando investigated photoinduced SET in FLP chemistry using resonance Raman spectroscopy aided by supporting DFT calculations.^37^ Resonance Raman spectroscopy of the archetypal PMes_3_/B(C_6_F_5_)_3_ system employing 457 nm excitation to overlap with the charge-transfer band showed the increase in signal intensity of specific signals compared to the normal Raman spectra (excitation at 1064 nm). The excitation in resonance with the charge-transfer band causes greater changes in the polarizability tensor of the vibrational modes associated with the EDA complex [PMes_3_, B(C_6_F_5_)_3_] and hence selective enhancement of Raman bands that were specifically attributed to the radical ion pair PMes_3_^•+^/B(C_6_F_5_)_3_^•–^. The author’s experimental results were supported by complementary DFT calculations, leading to the important conclusion that the enhancement of intensity in Raman signals originated from vibrations in both B(C_6_F_5_)_3_ and PMes_3_. The involvement of *both* components of the FLP system further confirms the occurrence of SET between Lewis base and acid upon irradiation of the charge-transfer band in the EDA.

As the concept of photoinduced SET in FLP chemistry is now well recognized within the literature as demonstrated here, the following sections highlight examples to showcase the potential applications accessible via these remarkable one-electron processes.

### 2.1 Application of Photoinduced Single-Electron Transfer: Utilization in Materials Science

**Figure 5:**
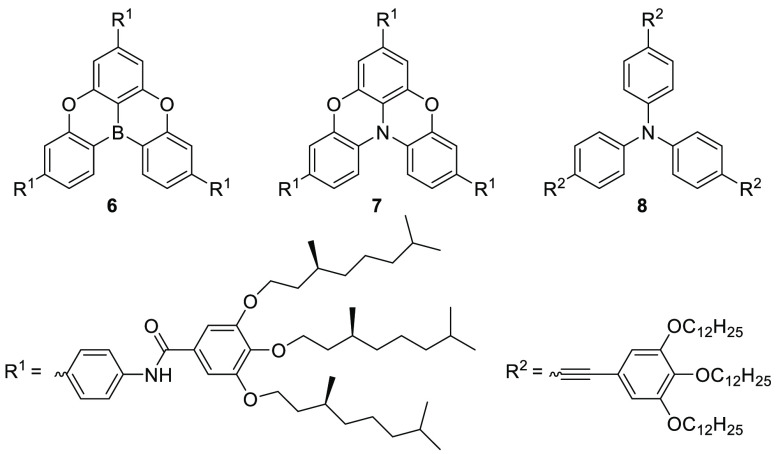
Copolymer formation resulting from stacking of amines and borane components. These copolymers were shown to undergo photoinduced single-electron transfer from the amine to the borane.

In search of multicomponent polymers with new properties, Meijer et al. reported on the photophysical properties of a copolymer consisting of stacked boranes and amines as monomers (Figure 5).^38,39^ For a combination of **6** and **7**, the authors observed a new absorption band in the UV–vis spectrum upon mixing the two components in decaline (λ_abs_ ∼500 nm).^38^ As neither of the individual LA or LB components absorb above 500 nm, this absorption band was assigned to the charge-transfer band of the encounter complex [**6**, **7**]. To explore the emission properties of the corresponding radical ion pair **7**^•+^/**6**^•–^ formed upon excitation, photoluminescence spectroscopy was employed. Excitation (λ_ex_ = 387 nm) of the FLP resulted in an additional emission band at 550 nm, which was not observed in corresponding measurements of the individual components even though both components have absorption bands at the selected excitation wavelength. The authors assigned this long wavelength emission to BET from the borane to the amine. The emission decay was characterized by two different lifetimes of ∼96 ns and ∼6 μs, which the authors assigned to decay of the singlet and triplet (caused by two electrons with the same spin in close proximity) states. Similar emission spectroscopy results were also reported by the authors for the combination **6** and **8**, although it should be noted that no UV–vis spectrum of the combination was reported to confirm the presence of a charge-transfer band.

**Figure 6:**
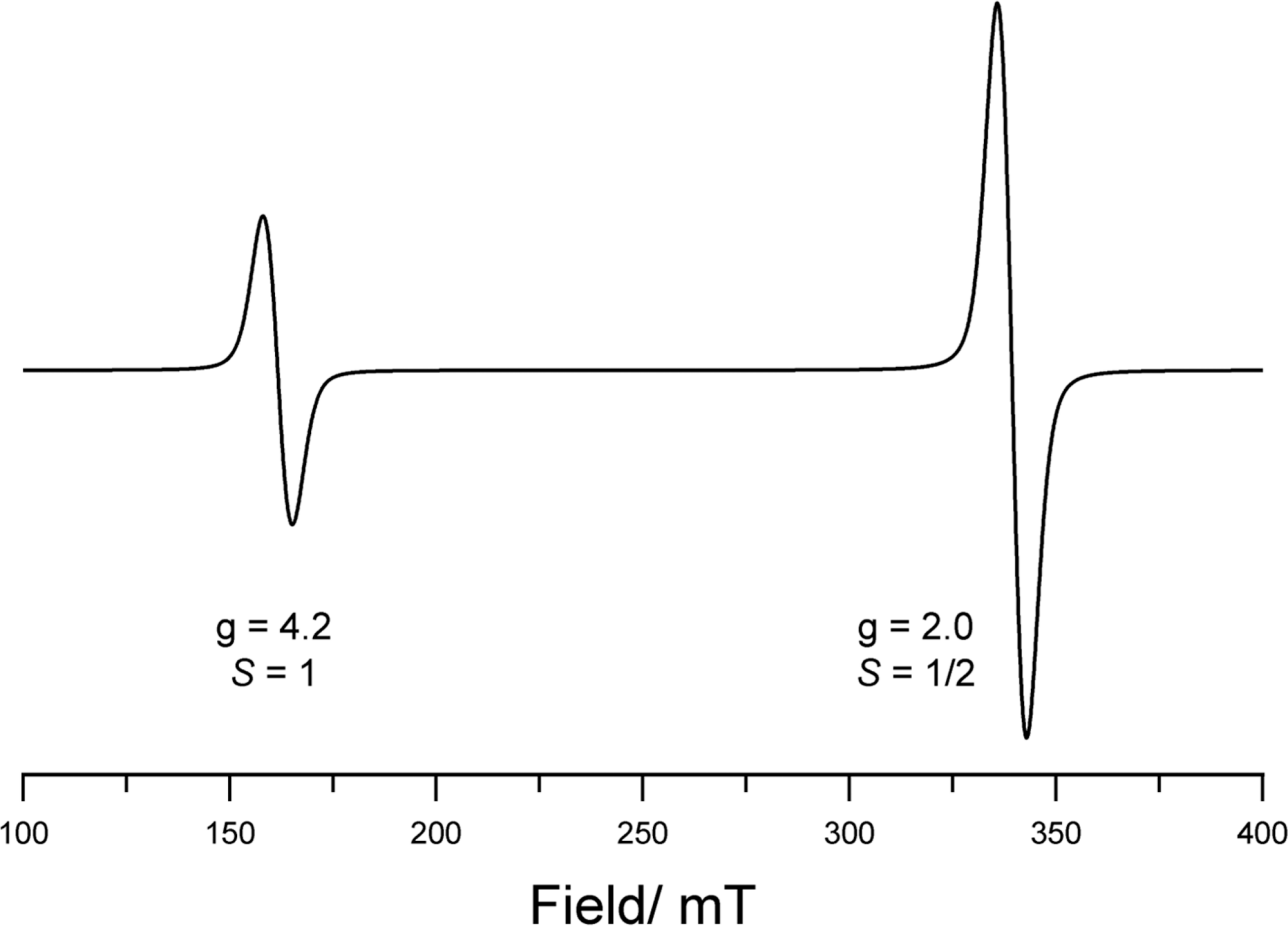
CW X-band EPR spectra of two independent EPR signals originating from low- and high-spin systems of a copolymer radical.

In a subsequent study, the authors used EPR spectroscopy to characterize the combination of arylamine **8** and B(C_6_F_5_)_3_ in a stacked copolymer.^39^ Complementary UV–vis spectroscopy showed no evidence of a charge-transfer band for this combination, although this could be due to the relatively low concentration of the components used in this study (both 25 μM). Despite this, two signals were observed in the low-temperature EPR spectra upon 320–390 nm irradiation of the copolymer, at *g* = 2.0 and *g* = 4.2 (Figure 6). The signal at *g* = 2.0 was weakly observed in the dark and grew in intensity upon irradiation, therefore indicating the formation of a low population of doublet-state isolated radical ions upon low-temperature excitation. The signal at *g* = 4.2 is characteristic of a triplet state (*S* = 1), arising from two coupled electrons of the same spin being localized in close proximity, likely arising from an N-to-B SET process. However, the individual components do also absorb the 320–390 nm light, therefore making it possible that these EPR results are due to absorption by one of the individual components.

**Scheme 5:**
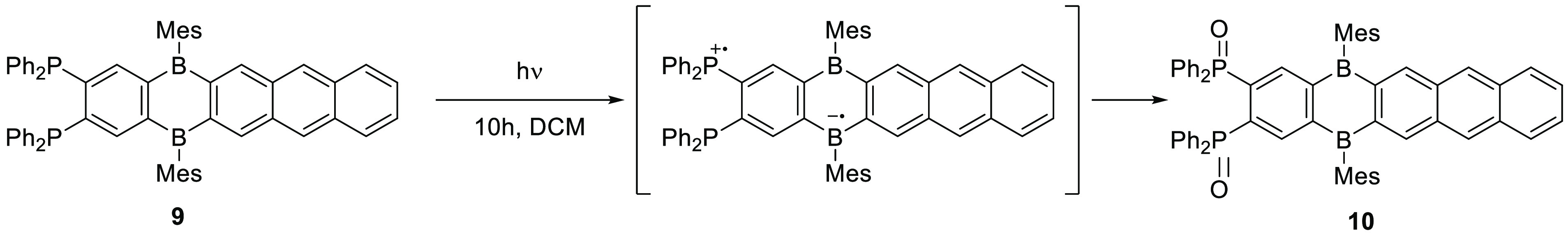
Intramolecular, Photoinduced SET from the Phosphines toward Boranes, Facilitating Oxidation toward Phosphine Oxides

In search of photoluminescent aryl boranes as novel optoelectronic materials, the Wagner group synthesized a doubly Ph_2_P-substituted dihydrodiborapentacene **9**.^40^ This compound is an air-stable solid in the dark, but when a DCM solution was exposed to ambient light, quantitative yield of the oxidized product **10** was obtained within 10 h (Scheme 5). The authors related this to a photoinduced, intramolecular charge transfer from the phosphine toward the borane backbone, yielding the corresponding radical ion pair. Phosphorus radical cations are known to react readily with oxygen to yield the corresponding phosphine oxide, just as the authors observed.^40^ The photoinduced SET is further supported by the calculation of the IE (5.50 eV) and EA (−2.79 eV) with DFT (SCF/B3LYP-D4/def2TZVP(-f) with SMD treatment of DCM).^40^ This results in the radical ion pair state calculated to be 2.71 eV (458 nm) higher in energy, which is comparable to the longest wavelength absorption band reported in benzene solution (470 nm, ε = 20800 mol^–1^ dm^3^ cm^–1^). TD-DFT also predicted the possibility of a photoinduced SET where electron density is moved from the phosphorus centers to the boranes (2.86 eV, 433 nm, *f*_osc_ = 0.186). It is important to note that the TD-DFT calculations (at the OT-LRC-ωPBEh/def2-TZVP level of theory with pt(SS+LR)-PCM treatment of toluene) also predicts other charge-transfer states in close proximity. For these excitations, the electron density is transferred from the anthracene toward the borane centers.

### 2.2 Photoinduced Single-Electron Transfer: Utilization in Synthesis

**Scheme 6:**
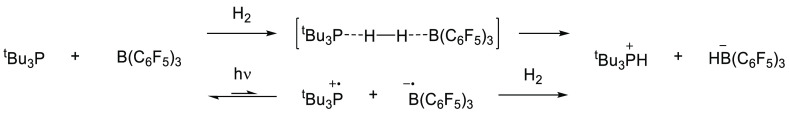
Activation of Dihydrogen by the FLP P^t^Bu_3_/B(C_6_F_5_)_3_ via Two-Electron (Top Reaction) or Single-Electron (Bottom Reaction) Mechanisms

As previously noted, dihydrogen activation was one of the first examples of an FLP-induced chemical modification.^2^ In this reaction, as clearly evidenced by Piers and co-workers, both radical (SET) and two-electron-transfer mechanisms can be envisioned (Scheme 6).^12^ In order to elucidate the prevalent reaction pathway, Slootweg et al. further investigated the PMes_3_/B(C_6_F_5_)_3_ combination.^17^ As shown above, the formation of the RIP PMes_3_^•+^/B(C_6_F_5_)_3_^•–^ via SET for this FLP pair occurs only under the influence of light. However, irradiation (534 nm, 2.2 W) was reported to have no significant influence on the reaction kinetics of H_2_ activation. Therefore, it was concluded that the radical pathway plays an insignificant role due to the transient nature of the RIP PMes_3_^•+^/B(C_6_F_5_)_3_^•–^ and rather the major contribution to this reaction chemistry is via a two-electron-transfer mechanism.

**Scheme 7:**
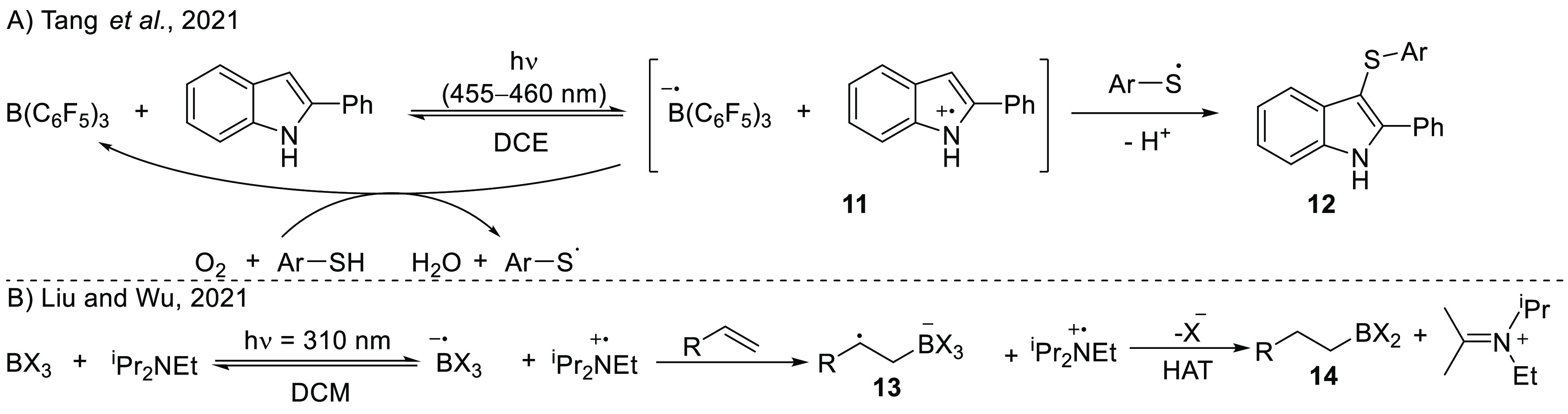
Synthetic Applications of the Photoinduced SET between Lewis Acid and Base: (A) Sulfenylation of 2-Phenyl Indole, Catalyzed by B(C_6_F_5_)_3_ via the Formation of an EDA Complex and Subsequent SET (Ar = *p*-^t^BuPh); (B) Hydroboration of Alkenes via the Photoinduced Radical Pair (X = Cl or Br)

Using photoinduced single-electron transfer employing B(C_6_F_5_)_3_ as the electron acceptor and catalyst, Tang et al. reported the sulfenylation of 2-phenyl indoles (Scheme 7A).^41^ UV–vis spectroscopy showed the formation of a charge-transfer band around 430 nm for the EDA [2-phenyl indole, B(C_6_F_5_)_3_]. Irradiation of this band with 455–460 nm light resulted in single-electron transfer from the indole to the borane, yielding the radical ion pair 2-phenyl indole^•+^/B(C_6_F_5_)_3_^•-^ (**11**), as confirmed by the observation of a signal in the EPR spectrum, characterized by *g*_iso_ = 2.00296 with no resolved hyperfine structure. The narrow spectral width of the signal (∼1.7 mT) precludes the assignment of this signal to the B(C_6_F_5_)_3_^•–^ anion (ΔB = 8 mT); therefore, this signal likely originates from the indole radical cation. The 2.4 V (517 nm) energy difference between indole oxidation (0.6 V vs Fc/Fc^+^ in acetonitrile^42^) and borane reduction (−1.79 V vs Fc/Fc^+^ in DCM^14^) confirms the feasibility of a photoinduced single-electron transfer. The authors proposed that following generation of the indole radical cation, addition of a thiyl radical and loss of a proton occurred, leading to the formation of the final product **12**. The formation of the thiyl radical is suggested to occur concomitantly during oxidation of the borane radical anion using oxygen as an oxidant. Furthermore, this process closes the borane catalytic cycle with selective oxidation of the reactive borane radical anion, achieving a high turnover number (TON) of 19 for B(C_6_F_5_)_3_.

Liu, Wu, and co-workers also used the photoinduced formation of a borane radical anion in the hydroboration of alkenes (Scheme 7B).^43^ The reaction begins with SET from ethyl di-*iso*-propyl amine to BX_3_ (with X = Cl or Br) to form the corresponding radical ion pair ^i^Pr_2_NEt^•+^/BX_3_^•–^, which was calculated via DFT to be 3.40 eV (78.3 kcal/mol) higher in energy than the closed shell pair, hence requiring photoinduced SET for its formation. TD-DFT calculations of Liu et al. also predict a charge-transfer band at 323 nm for SET from the amine to borane. After the formation of the RIP, the authors propose that the borane radical anion adds to the alkene, yielding radical intermediate **13**, based on more DFT calculations. After hydrogen atom abstraction from the amine radical cation and halogen loss from **13**, the final product **14** is obtained. To determine the proton source, deuterium-labeling experiments were performed using *d*_8_-styrene and *d*_2_-dichloromethane. For both deuterium sources, no *d*-incorporation was observed in the β-position of the product. Instead, the presence of an iminium cation was confirmed by observation of a resonance at 152.6 ppm in the ^13^C NMR spectra and by ESI-MS analysis (128.1435 (observed) vs 128.1434 (calculated)), indicating the amine radical cation to be the hydrogen atom source. Further mechanistic studies regarding halogen loss indicated the formation of BBr_4_^–^ by the observation of a peak at −24.32 ppm in the ^11^B-NMR spectrum. An inhibition of the reaction was observed in the presence of radical scavengers like 2,2,6,6-tetramethylpiperidine-1-oxyl (TEMPO) or 2,6-di*tert*-butyl-4-[(3,5-di*tert*-butyl-4-λ-1-oxidanylphenyl)methylidene]cyclohexa-2,5-dien-1-one (galvinoxyl), once more proving a radical mechanism.

**Scheme 8:**
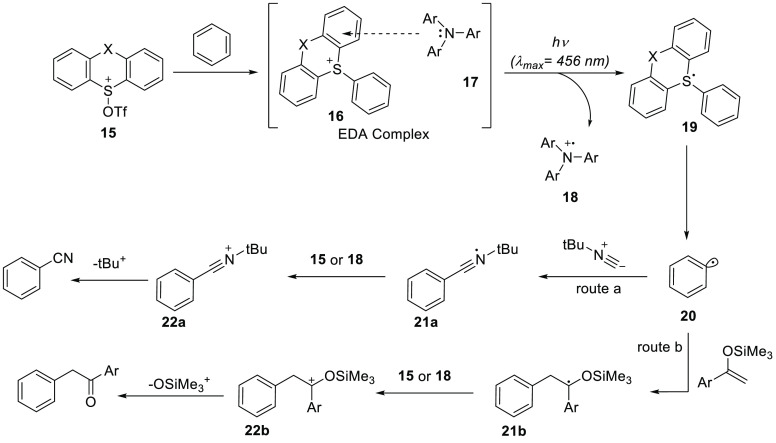
Photoinitiated Donor–Acceptor Pairs Catalyzed Site-Selective C–H Cyanation and Alkylation of Arenes X = O or single bond. Ar = 1-Naphthyl, phenyl, *para*-bromo phenyl or *para*-chloro phenyl.

Besides the typical borane Lewis acids as electron acceptors, there are also reports of using sulfonium salts as the acceptor in combination with, among others, amines or sulfur-based electron donors.^44^ A prime example is the C–H functionalization of arenes using triarylsulfonium salt **15**, as recently reported by Bednar et al. and Procter et al. (Scheme 8).^45,46^ Mixing **15** with the arene gave the adduct **16**, which together with the amine **17** formed EDA complex [**17**, **16**], which is characterized by a small red-shifted absorption in the UV–vis spectrum. The subsequent photoinduced SET (λ_em_ = 456 nm) led to the formation of both the amine radical cation **18** and the sulfur radical **19**, of which the latter undergoes homolytic bond cleavage to yield the sp^2^-arene radical **20**. Next, this radical is trapped by the nucleophile ^t^Bu-isocyanide (route a) or an enol silane (route b) to yield the functionalized arene radical **21**. Oxidation of this radical by the amine radical cation **18** or sulfonium cation **15** yields the corresponding arene cation **22**. The final product is obtained after elimination of the ^t^Bu cation (route a) or silyl cation (route b). With this one-pot procedure, the authors were able to obtain a wide range of α-arylated carbonyl compounds using route a^47,48^ and cyanated arenes with route b,^49,50^ which are hard to prepare in the absence of transition-metal catalysts, organometallic reagents, and toxic cyanides.

## 3 Thermal Single-Electron Transfer

**Scheme 9:**
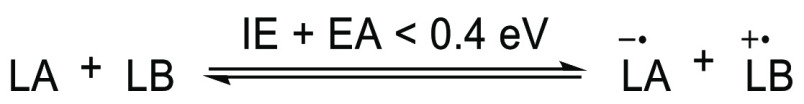
For Combinations of Lewis Acids (LA) and Bases (LB) with a Sum of the Electron Affinity and Ionization Energy, Respectively, Smaller than 0.4 eV, a Thermal SET Can Potentially Be Observed

As shown in section 2, for SET to be induced with visible light, the energy gap between the ionization energy of the Lewis base and the electron affinity of the Lewis acid should be in the range 1.5–3.1 eV. It is also possible to thermally induce SET by reducing the energy difference between IE and EA, and as such reducing the energy difference between the ground state EDA complex and the corresponding radical ion pair. This will lead to an equilibrium between the closed shell and radical states as shown in Scheme 9. Following the Boltzmann equation, for 0.06 M solutions (or 0.03 M of both donor and acceptor) an energy gap of ±0.4 eV (±9 kcal/mol) can already lead to detectable concentrations of the radical pair in EPR experiments^16^ since concentrations as low as the 10^–10^ M region can be detected.^51^ In the following sections several FLP and main group systems will be discussed to highlight the feasibility of a thermal SET.

### 3.1 Evidence of Thermal Single-Electron Transfer

**Scheme 10:**
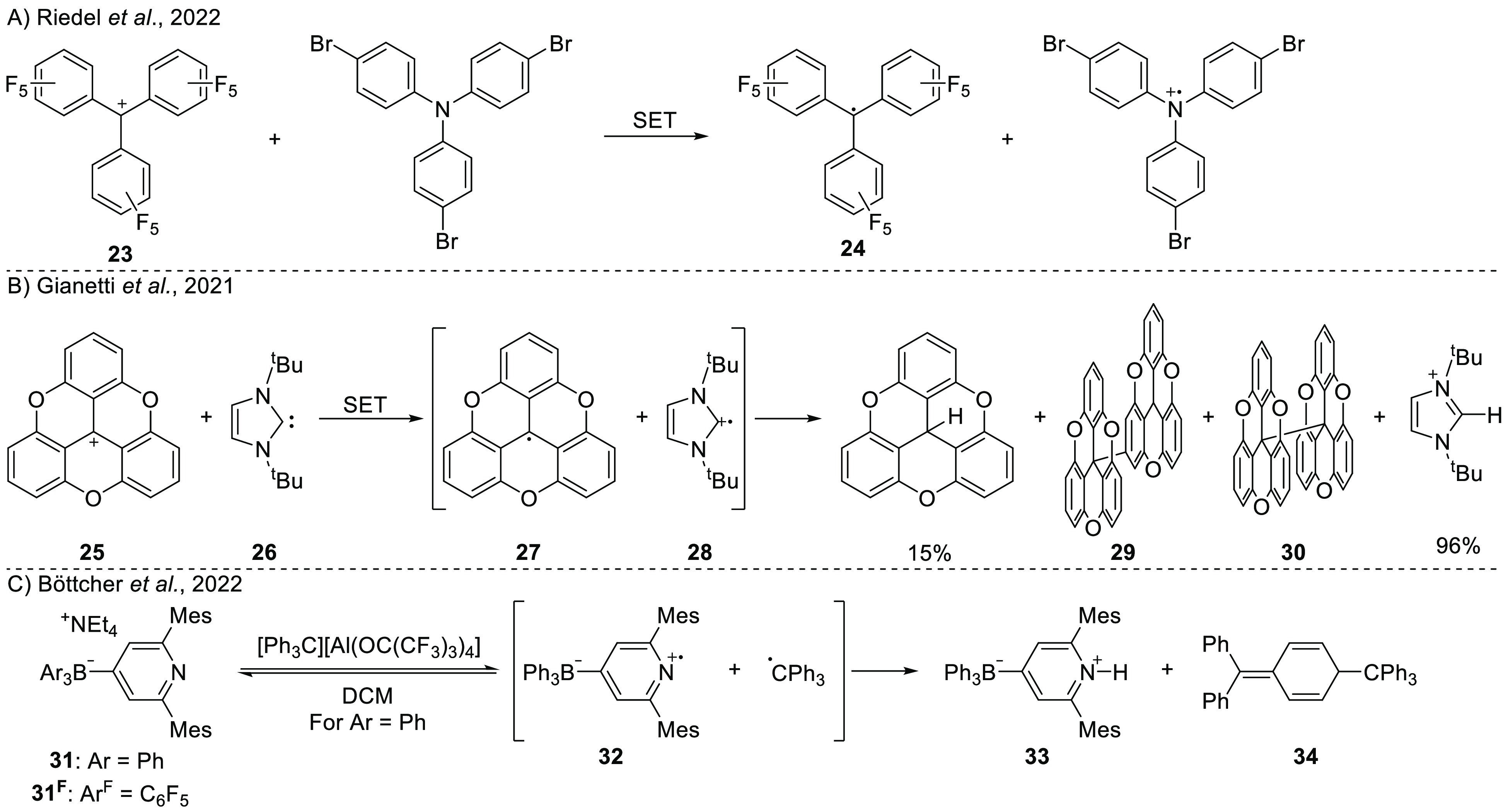
Selected Examples of Thermal SET in FLP Chemistry: (A) Perfluorinated Trityl Cation as an Electron Acceptor with a Triarylamine; (B) Thermal SET between TOTA^+^ and I^t^Bu and the Decomposition of Both Radicals; (C) SET Proposed to Occur upon Mixing a Boron-Substituted Pyridine and the Trityl Cation For the fluorinated pyridine **31**^**F**^ no reaction occurred.

Riedel et al. recently reported thermal SET using the fluorinated trityl cation [C(C_6_F_5_)_3_][Al(OTeF_5_)_4_] (**23**).^52^ This substituted carbon-based Lewis acid was determined to have an increased electron affinity compared to the nonfluorinated trityl cation (CPh_3_^+^) (−7.33 eV vs −5.86 eV, respectively). This is in agreement with trends in the oxidation potential, which increases from −0.11 V vs Fc/Fc^+^ in MeCN for CPh_3_^•^/CPh_3_^+^ to 1.11 V vs Fc/Fc^+^ in *o*-difluorobenzene for C(C_6_F_5_)_3_^•^/C(C_6_F_5_)_3_^+^. Addition of tris(*p*-bromophenyl)amine (0.74 V vs Fc/Fc^+^ in DCM) to the fluorinated trityl cation **23** led to the formation of the radical pair (*p*Br-Ph)_3_N^•+^/C(C_6_F_5_)_3_^•^ (**24**), as directly evidenced by the observation of both radical ions in the EPR spectrum as broad, featureless overlapping singlets characterized by *g*_iso_ ∼ 2.0031 and *g*_iso_ ∼ 2.012 for the perfluorinated trityl and arylamine radical cation, respectively (Scheme 10A). As the redox potential of the fluorinated trityl cation is 0.4 V higher than the redox potential of the amine, thermal SET is indeed possible for this combination of electron donor and acceptor.

**Figure 7:**
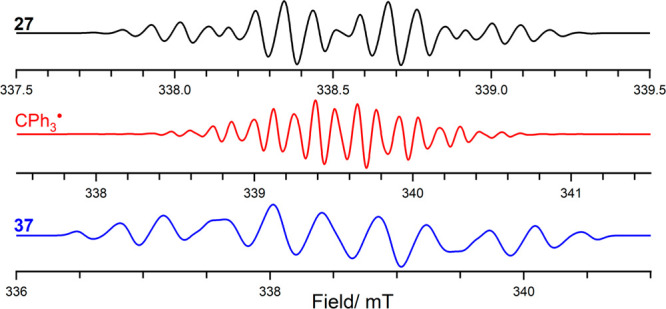
CW EPR spectrum of **27**, trityl **CPh**_**3**_^•^ radical, and **37**; simulated using values listed in Table 1.

The group of Gianetti studied the reactivity between trioxatriangulenium (TOTA^+^, **25**) with 1,3-di-*tert*-butylimidazol-2-ylidene (I^t^Bu, **26**; Scheme 10B).^53^ Mixing the two compounds in toluene or acetonitrile gave directly a green solution, which is an indication of possibly a SET event. The corresponding radicals TOTA^•^**27** and I^t^Bu^•+^**28** were not observed via EPR spectroscopy, either directly or via trapping experiments with TEMPO and benzoyl peroxide. However, the formation of TOTA-dimers **29** and **30** as components of the product mixture suggested the presence of radical intermediates. The calculated electron affinity of TOTA^+^ (−7.41 eV, PCM/CAM-B3LYP/6-311G(d,p) in acetonitrile) and the ionization energy of I^t^Bu (5.59 eV) support the premise that a thermal SET mechanism is possible. As a final proof for the occurrence of a thermal SET, the authors studied the properties of the TOTA dimer. Upon measuring a solution of the dimer in *m*-xylene, the TOTA monomer radical could be observed at temperatures above 340 K with EPR spectroscopy and was identified by a multiline EPR signal characterized by *g*_iso_ = 2.005, *a*_iso_(^1^H_*n*=6_) = 0.091 mT and *a*_iso_(^1^H_*n*=3_) = 0.328 mT (Figure 7).

The reduction of metals by pyridine scaffolds was observed by Böttcher et al. during their attempts of coordinating *para*-borane-substituted pyridines **31** and **31**^**F**^ (Scheme 10C) to metal centers.^54^ To study the SET process in more detail, they mixed **31** with the trityl cation (CPh_3_^+^) in DCM. The obtained products were the protonated pyridine **33** and Gomberg’s dimer, **34**. Detection of the Gomberg dimer indicates the occurrence of SET, and indeed the redox potentials (0.56 V vs Fc^+^/Fc for **31**, and 0.66 V vs Fc^+^/Fc for the trityl cation) show that a thermal SET is feasible. The authors proposed that hydrogen atom abstraction from the solvent by the pyridine radical cation **32** proceeds following the SET event. No evidence for SET was observed using the fluorine-substituted **31**^F^ analogue, which is in-line with the higher oxidation potential (1.32 V vs Fc^+^/Fc) of this borate.

**Scheme 11:**
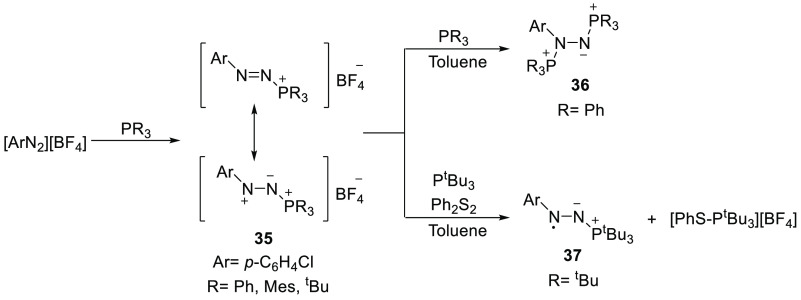
Reactivity of FLPs Involving a Nitrogen-Centered Lewis Acid Ar = *p*-C_6_H_4_Cl.

Although the broad field of FLP chemistry is mainly based on the use of B(C_6_F_5_)_3_ and related electrophilic boranes, in addition to a small selection of Al-based examples, Stephan et al. have recently reported an FLP system featuring a Lewis acidic nitrogen center to uncover the use of new Lewis acids in FLP chemistry.^55^ Treatment of PR_3_ (R = Ph, ^t^Bu, or Mes) with a diazonium salt afforded the diazonium cation **35**,^16,17^ featuring a Lewis acidic nitrogen center (Scheme 11). The PPh_3_-containing diazonium salt directly undergoes a second phosphine addition to give the stable bis-phosphine [(*p*-ClC_6_H_4_)N(PPh_3_)N(PPh_3_)]^+^ species **36**. The use of the more bulky phosphines P^t^Bu_3_ and PMes_3_ prevents a second addition, making the observation of **35** feasible.

Interestingly, in the presence of a disulfide, the combination of the diazonium Lewis acid–P^t^Bu_3_ adduct **35** and a second equivalent of P^t^Bu_3_ is able to cleave the disulfide bond, yielding [PhS–P^t^Bu_3_][BF_4_] as evidenced by a chemical shift of 85 ppm in the ^31^P(^1^H) NMR spectrum. A color change of the reaction solution from purple to an intense red upon addition of the disulfide indicated the formation of the stable radical species **37** (Scheme 11). This was confirmed upon observation of a paramagnetic species in the EPR spectrum (Figure 7), characterized by multiple hyperfine interactions [*a*_iso_(^31^P) = 0.88 mT, *a*_iso_(N_1_) = 0.42 mT, *a*_iso_(N_2_) = 0.36 mT, *a*_iso_(H_ortho_) = 0.36 mT, and *a*_iso_(Cl) = 0.28 mT] (Figure 7).

Independent generation of the radical **37** from the diazonium-phosphine adduct **35** with various reducing agents (Cp_2_Co, potassium, and PhSNa) suggest that **35** is reduced during the cleavage of the disulfide bond. The reported redox potential of the diazonium-phosphine adduct **35** (*E*_red_ = −0.91 V vs Fc/Fc^+^ in MeCN) is in accordance with a possible thermal SET using P^t^Bu_3_ as the oxidant (*E*_ox_ = 0.90 V vs Fc^+^/Fc in MeCN) yields an energy gap of Δ*E*_ox-red_ = −0.01 V.^13^

### 3.2 Reactions with a Thermal Single-Electron-Transfer Step

**Scheme 12:**
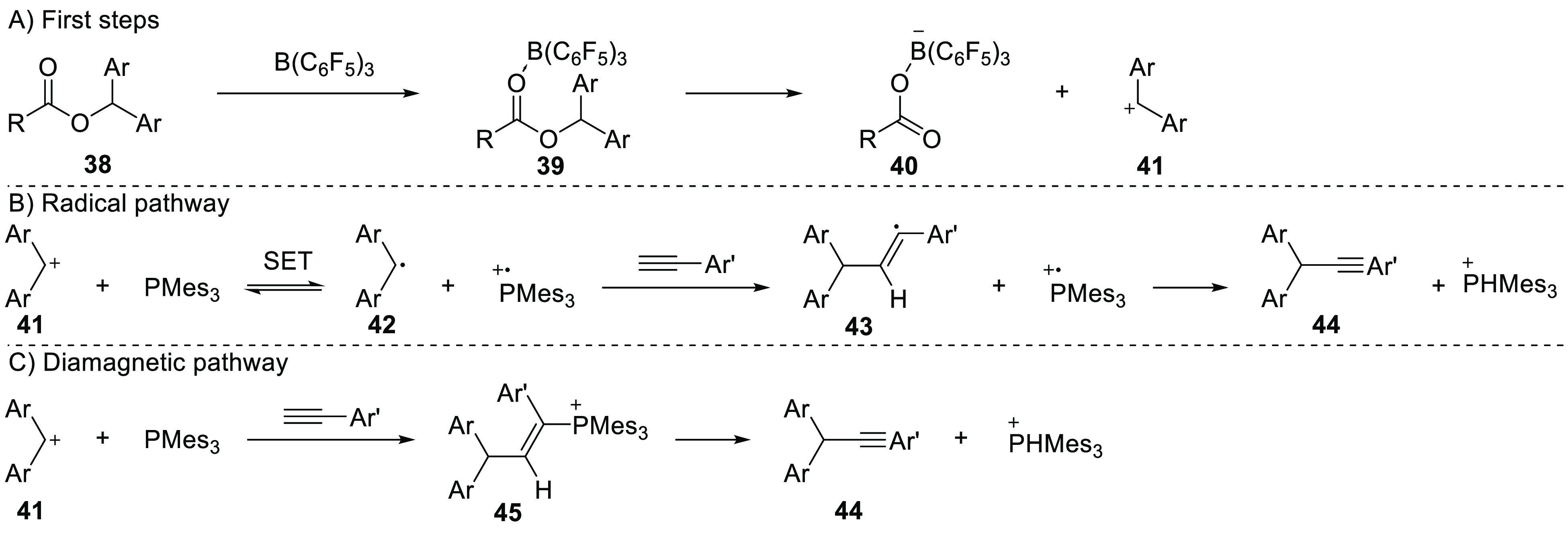
Proposed Mechanism for the C–C Bond Formation between a Diarylester and Phenylacetylene Using the PMes_3_/B(C_6_F_5_)_3_ FLP: (A) Formation of the Diaryl Carbocation; (B) Radical Pathway Leading to the Product; (C) Diamagnetic Pathway Leading to the Cross-Coupled Product

Melen’s group reported on the use of diaryl esters (**38**, Scheme 12) for the formation of C–C bonds in the presence of the PMes_3_/B(C_6_F_5_)_3_ FLP system, providing both experimental and computational details to support proposed reaction mechanisms.^56,57^ The reaction proceeds initially with the coordination of B(C_6_F_5_)_3_ to the ester **38** (Scheme 12A) to form adduct **39**, followed by heterolytic cleavage of the C–O bond, yielding the borane **40** and carbocation **41**. The carbocation can undergo a reversible one-electron reduction by PMes_3_ to the radical **42** (Scheme 12B), as evidenced by room temperature EPR spectroscopy (Figure 9). The authors found that this radical state is only 6.9 kcal/mol (0.30 eV) higher in energy than the ion pair, making a thermally induced SET indeed feasible. The absence of the carbon radical **40** was justified by its quick decomposition. It should be noted that Melen et al. were able to observe a weak signal in the EPR spectrum assigned to a more sterically encumbered carbon radical for a related system, confirming the formation of a radical pair.^56^

Continuing from the equilibrium between cation **41** and radical **42**, the reaction can proceed via two alternative pathways.^57^ The radical mechanism continues from the radical **42** by addition to the alkyne to give **43** as an intermediate, as shown in Scheme 12B. After hydrogen atom abstraction by the phosphine radical cation, the final product **44** is obtained. Alternatively, the diamagnetic pathway proceeds with the addition of both the carbocation **41** and phosphine to phenylacetylene (Scheme 12C). The product **44** is obtained after elimination of PMes_3_ from the intermediate **45**. To probe the operative mechanism, the authors determined the Hammett parameter ρ by doing competition experiments with *para*-substituted phenyl acetylenes. They found values for ρ of −6.6 ± 1.7 experimentally and −5.7 ± 0.8 computationally, which indicates the buildup of a positive charge during the reaction. These results are consistent with the diamagnetic mechanism. DFT calculations of the energy surface show that for electron-donating *para*-substitutions on the phenylacetylene, the reaction barrier of the radical mechanism is up to 18.8 kcal/mol (for *p*-NMe_2_ phenyl acetylene) higher in energy than the barrier in the diamagnetic pathway. The difference between the radical and diamagnetic mechanism is however smaller when utilizing electron-withdrawing substituents. For example, the difference is only 0.7 kcal/mol for *p*-NO_2_ phenyl acetylene, indicating that the radical mechanism can be (partly) operative with electron-poor substrates.

**Scheme 13:**
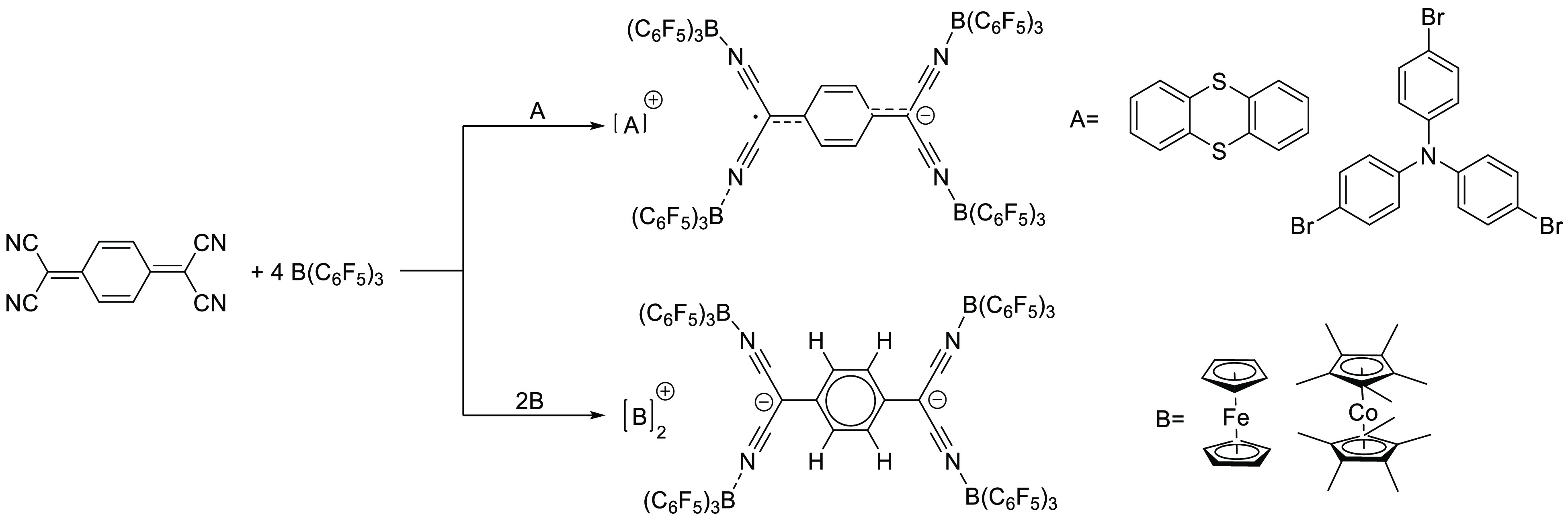
Oxidation Reactions of the Combination of TCNQ and B(C_6_F_5_)_3_

Very recently, Malischewski’s group increased the EA of free TCNQ from experimentally determined 3.38 eV for the first oxidation to 6.04 eV upon addition of four equivalents of B(C_6_F_5_)_3_, as determined by DFT calculations at the B3LYP-D3(BJ)/Def2-SVP level of theory.^84^ Also the EA of the second oxidation of 3.21 eV was found to be more facile than the first oxidation of free TCNQ. The authors showed that TCNQ-(B(C_6_F_5_)_3_)_4_ is capable of oxidizing both *p*BrPh_3_N and thianthrene in DCM (upper reactions in Scheme 13) by observation of two partly overlapping singlets in either case. In the case of *p*BrPh_3_N, the authors assigned the singlet at *g*_iso_ = 2.0143 to the *p*BrPh_3_N^•+^ radical cation and for the TCNQ-(B(C_6_F_5_)_3_)_4_^•–^ radical anion a signal at *g*_iso_ = 2.0026. In the case of employing thianthrenium, the radical anion was observed at the same position and the thianthrenium radical cation at *g*_iso_ = 2.00788. Independent generation of the monoanion with ferrocene showed a broad singlet at *g*_iso_ = 2.00238, confirming the assignment of the radical anion. Furthermore, for both *p*BrPh_3_N and thianthrenium the authors were able to obtain crystal structures in combination with TCNQ-(B(C_6_F_5_)_3_)_4_, which showed the coordination of the boranes to each of the cyanides of TCNQ.

Formation of the dianion [TCNQ-(B(C_6_F_5_)_3_)_4_]^2–^ was achieved with ferrocene (Fc) and decamethylcobaltocene (Cp*_2_Co) (bottom reactions in Scheme 13).^58^ X-ray diffraction confirmed again the coordination of the boranes to the cyanides of TCNQ. The dianion also made it feasible for the authors to measure the oxidation potential of the dianion and monoanion. The first oxidation in DCM of [Cp*_2_Co]^+^_2_[TCNQ-(B(C_6_F_5_)_3_)_4_]^2–^ was found at −0.065 V vs Fc/Fc^+^ and the second oxidation at +1.226 V vs Fc/Fc^+^. Compared to the first reduction potential of −0.30 V vs Fc/Fc^+^ and −0.88 V vs Fc/Fc^+^ for the second reduction of TCNQ, this shows a significant increase of oxidation power, in-line with the found EAs.

**Scheme 14:**
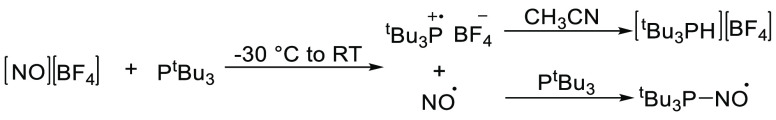
Single-Electron Transfer of P^t^Bu_3_ with [NO][BF_4_]

**Figure 8:**
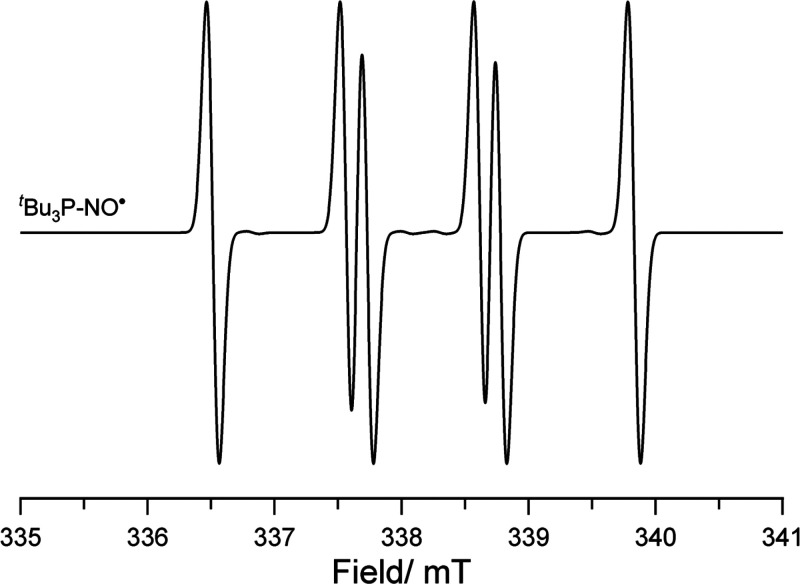
CW X-band EPR spectra of substituted nitroxyl; simulated using values listed in Table 1.

Slootweg et al. reported the one-electron oxidation of P^t^Bu_3_ by single-electron transfer (SET) using the strong oxidant nitrosonium salt [NO][BF_4_] [(NO^+^/NO^•^) = 0.87 V vs Fc/Fc^+^ in MeCN],^58^ generating [^t^Bu_3_PH][BF_4_] as the major product (Scheme 14).^13^ The reaction was predicted to proceed through the formation of the radical salt intermediate [P^t^Bu_3_]^•+^[BF_4_]^−^ as the oxidation potential of P^t^Bu_3_ (0.90 V vs Fc/Fc^+^ in MeCN) is in the range of a thermal SET. The generated phosphorus radical cation readily abstracts a proton from the acetonitrile solvent and subsequent decomposition affords the phosphonium borate product. Using EPR studies, a small side reaction was found to occur as the authors found a six-line pattern characterized by *g*_iso_ = 2.0071, *a*_iso_(^14^N) = 1.05 mT, and *a*_iso_(^31^P) = 1.21 mT (Figure 8). They assigned this to the formation of the nitrosyl–phosphine adduct ^t^Bu_3_P–NO^•^, which can be established by trapping the in situ generated NO^•^ by residual P^t^Bu_3_.

**Scheme 15:**
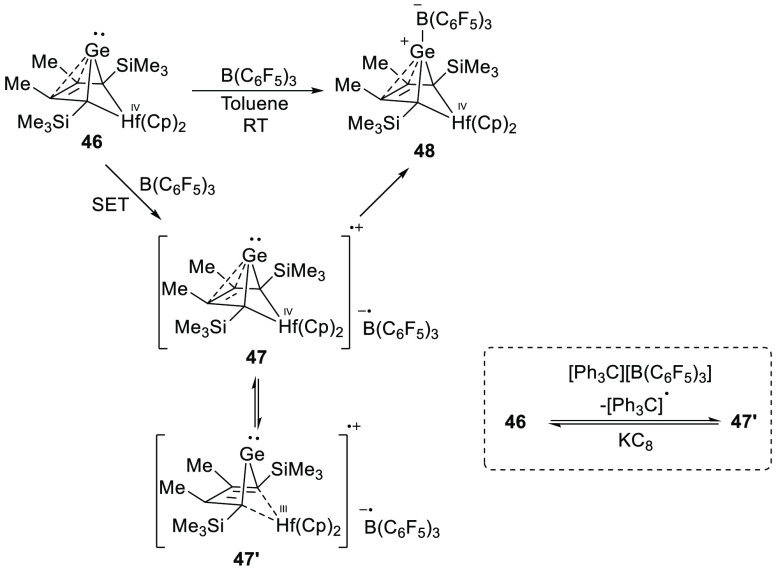
Proposed Generation of Radical Ion Pair Species from a Germanium-Centered Lewis Base with B(C_6_F_5_)_3_ and a Control Reaction Using the Trityl Cation as a One-Electron Oxidant for the Lewis Base

**Figure 9:**
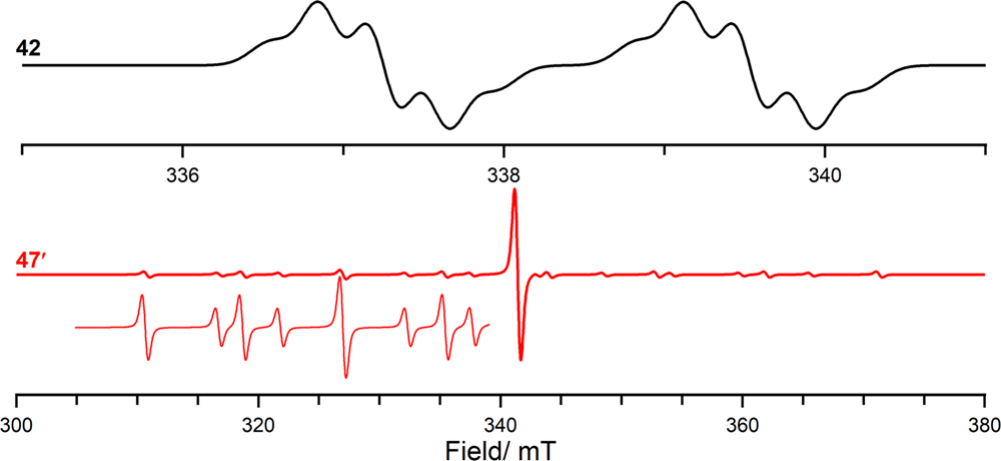
CW EPR spectrum of **42** and **47′**; simulated using values listed in Table 1.

An exciting example of SET has recently been reported by Müller et al., in which they described the coordination of B(C_6_F_5_)_3_ to the germanium-centered Lewis base **46** (Scheme 15).^59^ During the reaction, the toluene solution turns into a deep purple color (λ_max_ = 544 nm), eventually turning colorless when the reaction is completed. Room temperature EPR spectra were recorded on reaction aliquots during the course of the reaction, in which the authors observed the formation of two different radical species (Figure 9). The first paramagnetic species was identified as the hafnium-based radical cation **47′** (*g*_iso_ = 1.9881, *a*_iso_(Hf) = 8.5 mT), which the authors propose to be in equilibrium with **47**. The authors propose that the formation of **47** occurs from **46** by a SET toward B(C_6_F_5_)_3_. Cyclic voltammetry showed indeed an irreversible oxidation ability of compound **46** (−0.51 V vs Fc^+^/Fc). Furthermore, oxidizing **46** with [Ph_3_C][B(C_6_F_5_)_4_] showed the formation of the same hafnium radical **47′** that could be detected together with the trityl radical, showing the occurrence of a SET with **46** as the reductant. The radical **47′** was also observed with EPR spectroscopy when a silyl cation was employed as the oxidant.

Besides the hafnium radical **47′**, the authors observed a second, featureless radical in the EPR spectrum when performing the reaction. They assigned it to the formation of the B(C_6_F_5_)_3_^•–^ radical anion and also proposed that this radical is responsible for the absorption at 544 nm, even though they mention that the B(C_6_F_5_)_3_^•–^ radical anion is highly unstable (*t*_1/2_ ≈ 5–10 min at 0 °C in THF).^19^ Furthermore, based on the reported reduction potential of B(C_6_F_5_)_3_ (−1.79 V vs Fc/Fc^+^ in DCM), a thermal SET seems unlikely due to the large energy gap (approximately 1.3 V). Therefore, we note here that a photoinduced SET cannot be excluded, where the observed deep purple color of the reaction mixture stems from the EDA complex [**46**, B(C_6_F_5_)_3_] that features a charge-transfer band in the UV/vis spectrum at λ_max_ = 544 nm. A similar misinterpretation was found for the observed purple color in the case of PMes_3_/B(C_6_F_5_)_3_ in chlorobenzene.^33^ For this FLP combination, the purple color was first thought to be indicative of the presence of radicals, but has since been shown to arise from the formation of the EDA complex [PMes_3_, B(C_6_F_5_)_3_].^16^

## 4 Consequences of Radical Decomposition in Radical Pairs Generated via SET

**Scheme 16:**
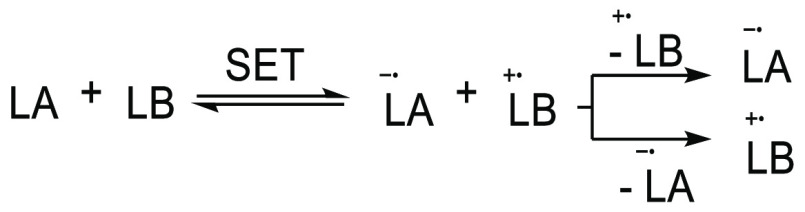
After the Formation of a Lewis Acid (LA) and Lewis Base (LB) Radical Ion Pair, Decomposition of Either Intermediate Will Lead to an Increased Concentration of the Other Radical

There are cases where a thermally induced SET can be observed even though the energy gap between the closed shell state and the radical (ion) pair is more than 0.4 eV. This can be attributed to the subsequent reaction of one of the radicals in a consecutive step (for example, hydrogen atom abstraction), as illustrated in Scheme 16. Further involvement of the radical in subsequent reactions will shift the SET equilibrium toward the radical products and thus increase the concentration of the more persistent radical, according to Le Chatelier’s principle.^60^ The consequence of this reactivity is that only one of the two radicals can be directly observed, instead of observing both radicals simultaneously as would be the case if both radicals are persistent. Furthermore, the decomposition of one of the two radicals can also occur after a photoinduced SET. BET is then not feasible anymore, leading to an increasing concentration of the persistent radical upon prolonged irradiation. The next sections contain reports where the decomposition of either of the formed radicals is required to observe radicals or where this is utilized in synthesis (Scheme 16).

### 4.1 Observations of Thermal Single-Electron Transfer after Radical Decomposition

**Scheme 17:**
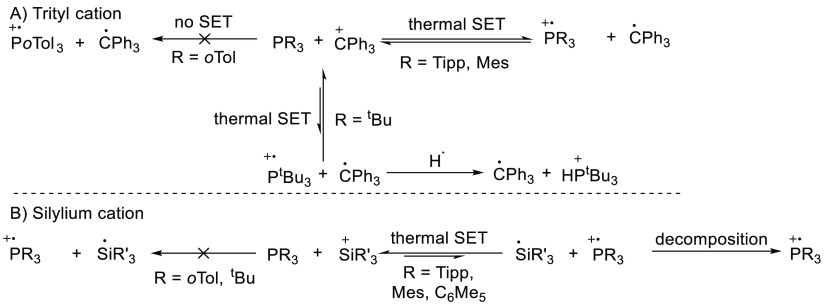
Reactivity of Several Phosphines with Different Electron Acceptors: (A) Trityl Cation As Electron Acceptor; (B) Silyl Cations as Electron Acceptors Tipp = 2,4,6-triisopropylphenyl. SiR_3_ = Si(C_6_Me_5_)_3_, Si^i^Pr_3_Si, Si^t^BuMe_2_, or SiEt_3_.

Klare et al. and Schilter et al. studied the reactivity of [Ph_3_C][B(C_6_F_5_)_4_] with different phosphines (Scheme 17A).^20,61^ Both tris-2,4,6-tri*iso*-propylphenyl phosphine (PTipp_3_) and PMes_3_ led to a SET event as both phosphine radical cations were observed by EPR (see Table 1 for details). The sum of the electron affinity and ionization energies were calculated as 0.07 and 0.33 eV (SCRF/M06-2*X*/6-311+G(d,p) in chlorobenzene), respectively, indicating that a SET thermal process was feasible for these phosphine derivatives. Later, Slootweg and co-workers showed that this is indeed the case as the radicals are formed in the dark.^16^ For P^t^Bu_3_, the sum of the electron affinity and ionization energy is 0.67 eV, which is typically too large to observe a thermal SET with EPR.^20^ However, the authors reported the presence of the trityl radical in the EPR spectrum. The ^31^P(^1^H) NMR spectrum showed the formation of tri-*tert*-butyl phosphonium salt, which was postulated to be formed by hydrogen abstraction by the phosphine radical cation. Therefore, it was postulated that decomposition of the phosphine radical cation leads to an increased concentration of the more persistent trityl radical, and hence increases the likelihood for experimental observation with EPR spectroscopy. The authors also studied the combination [Ph_3_C][B(C_6_F_5_)_4_] with P*o*Tol_3_, for which the calculated energy gap (1.04 eV) completely precluded a thermal SET, as confirmed by the absence of detectable radicals in the EPR spectrum; hence, no radicals were available for subsequent reactivity.

Klare and Müller et al. have also studied silylium ions as the choice of electron acceptor (Scheme 17B).^20^ The silylium ions are weaker electron acceptors than the trityl cation, resulting in the IE/EA sum to be larger than 0.4 eV in all cases, which normally would make the observations of radicals with EPR spectroscopy impossible. However, the obtained silyl radicals are known to be highly unstable and can quickly decompose upon formation,^62^ pushing the equilibrium in favor of the phosphine radical cation. We suggest that this decomposition therefore results in the enhanced ability of the authors to observe in certain cases the partner phosphine radical cation. For example, for the silylium cation [(Me_5_C_6_)_3_Si][B(C_6_F_5_)_4_] in combination with PTipp_3_ or PMes_3_, the IE/EA sum is 0.92 and 1.18 eV, respectively, but the phosphine radical cation is clearly visible in the resulting EPR spectrum.^20,61^ In contrast, no EPR signals were observed for either the P^t^Bu_3_ and P*o*Tol_3_ systems, which the authors explained as arising from the energy gap being too large to be thermally overcome for the radical state to be accessed. Furthermore, the phosphine radical cations of P^t^Bu_3_ and P*o*Tol_3_ are both thermally unstable, further reducing the possibility of observation of any radicals via EPR spectroscopy as both components, the electron donor and acceptor, are unstable in their corresponding radical state.

**Scheme 18:**
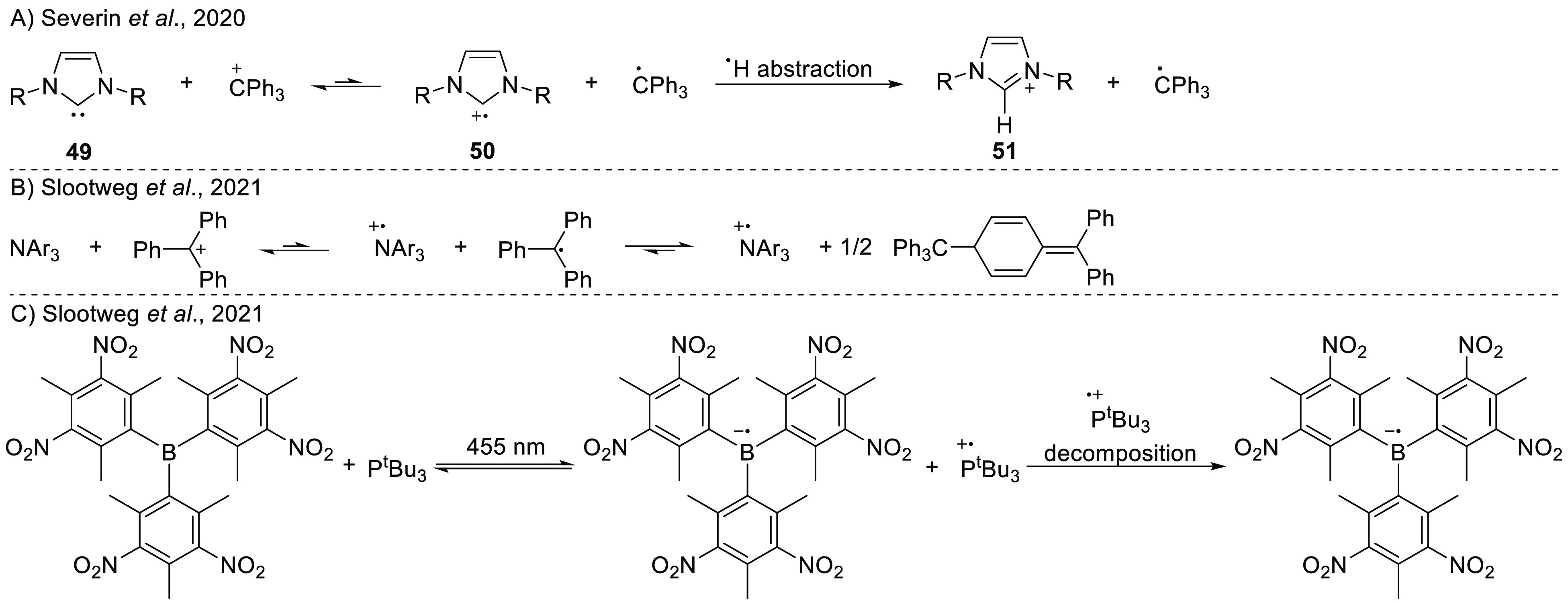
(A) Reduction of the Trityl Cation by NHCs and Subsequent Decomposition of the NHC Radical Cation; (B) Equilibria between a Triarylamine and the Trityl Cation, Leading to Observable Quantities of the Amine Radical Cation; (C) SET between P^t^Bu_3_ and a Borane, Affording a Thermally Stable Borane Radical Anion R = Dipp (2,6-di-*iso*-propylphenyl), ^t^Bu, or Mes (2,4,6-trimethylphenyl). Ar = Ph or *para*-tolyl (*p-*Tol).

Severin et al. reported the use of N-heterocyclic carbenes (NHCs) as electron donors in toluene.^63^ Upon mixing the NHC IDipp (**49** with R = 2,6-di-*iso*-propylphenyl) and the trityl cation (see Scheme 18A), a short-lived purple solution was obtained. Both the resulting room temperature EPR spectrum and UV–vis spectroscopy measurements (revealing an absorption band at 343 nm) showed evidence of the trityl radical. The IDipp radical cation **50** was not observed by EPR, presumably due to its facile decomposition. On the other hand, the authors were able to assign an absorption band at 591 nm to the IDipp radical cation **50**, which was observed during the early stages of the reaction. The authors supported this assignment by independently oxidizing IDipp with [NO][PF_6_], which showed the same short-lived purple color as observed for the FLP solution. Trapping the IDipp radical cation **50** with hydrogen atom donors (Ph_3_SnH and THF) led to the formation of the imidazolium salt **51**, again supporting an accessible SET pathway. Similar behavior was reported for the I^t^Bu and IMes NHC derivatives (**49**, where R = ^t^Bu or Mes). Calculations by Gianetti et al. determined that the radical pair is 0.94 eV higher in energy for I^t^Bu (**49** with R = ^t^Bu)/CPh_3_^+^ (PCM/CAM-B3LYP/6-311G(d,p) in acetonitrile).^53^ Typically, this is too high in energy to observe any radical species, but we suggest that the rapid decomposition of the NHC radical cation **50** allows a buildup in concentration of the trityl radical, making its experimental observation with EPR spectroscopy (*g*_iso_ = 2.0025, *a*_iso_(*o*-H) = 0.26 mT, *a*_iso_(*m*-H) = 0.11 mT, *a*_iso_(*p*-H) = 0.28 mT) possible.

For a SET between N*p-*Tol_3_ or NPh_3_ and [CPh_3_][B(C_6_F_5_)_4_] (IE/EA sum = 0.43 and 0.7 eV, respectively, at SCRF/ωB97X-D/6-311+G(d,p) in toluene), the radical pair state is too high in energy to observe any radical formation by EPR.^16^ However, Slootweg et al. showed that the dimerization of the trityl radical toward Gomberg’s dimer drives the equilibrium toward the radical state (Scheme 18B). This increases the concentration of the amine radical cations, making it possible to observe these radicals in toluene solution at room temperature with EPR spectroscopy.

### 4.2 Formation of a Single Radical Species after Photoinduced Single-Electron Transfer

It should again be noted that decomposition of one of the radicals can occur after photoinduced SET. Slootweg et al. observed this for the combination P^t^Bu_3_/B(NO_2_-Mes)_3_ in DCM (Scheme 18c), for which the corresponding radical ion pair P^t^Bu_3_^•+^/B(NO_2_-Mes)_3_^•–^ is 2.91 eV (67.1 kcal/mol) higher in energy than the ground state (SCRF/ωB97X-D/6-311+G(d,p) in toluene).^16^ Upon irradiation of a mixture of both the donor and acceptor in DCM for 3 h, a red solution was obtained, and the presence of the thermally stable borane radical anion was confirmed by a signal (with some hyperfine features, presumably due to coupling with the boron center) in the EPR spectrum. The red color of the borane radical anion solution was persistent, as BET was prevented by the rapid decomposition of the phosphine radical cation.

### 4.3 Utilization of Radical Reactivity in Synthesis

**Scheme 19:**
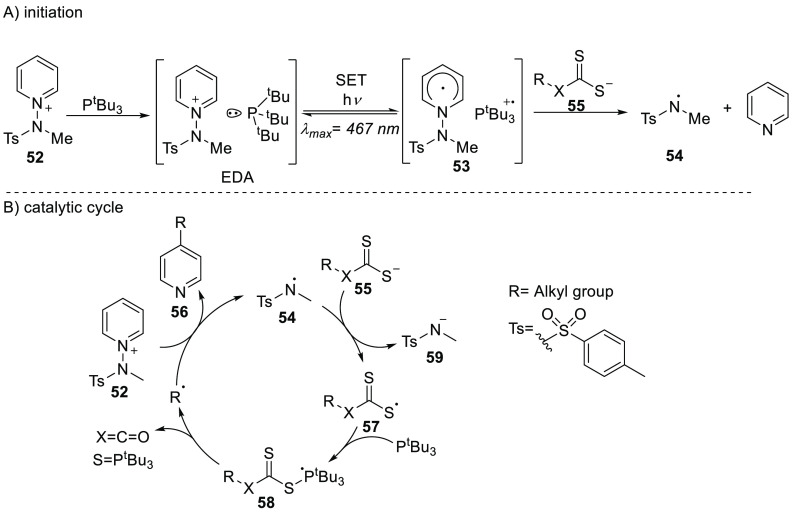
Alkylation of Pyridines via Photoinduced SET between a Pyridinium Salt **52** and Xanthate Anion **55**, Starting with (A) Initiation and (B) the Catalytic Cycle X = O or S.

Hong’s group reported recently the C–H functionalization of pyridinium salts using alcohols and thiols.^64^ This reaction was proven to proceed via both a dark reaction and a photoinduced pathway, as evidenced by the results that a 72% yield was obtained in the absence of irradiation, which improved slightly to 84% upon irradiation (λ = 467 nm). The reaction started with an initiation, which the authors proposed for the dark reaction to be a SET between P^t^Bu_3_ and the pyridinium salt **52** (Scheme 19A). This yielded a radical pair consisting of P^t^Bu_3_^•+^ and radical **53**. Upon the formation of radical **53**, homolytic bond cleavage occurred toward aminyl radical **54** and pyridine. The photoinduced reaction was also proposed to be initiated by the formation of radical **53**. The authors showed the presence of a new broad absorption band around 550 nm, which was formed upon mixing pyridinium salt **52** and the *in situ* formed xanthate anion **55**. Irradiation of this band led to SET and via the formation of radical **53** toward aminyl radical **54**, which was also obtained in the dark initiation reaction.

The authors proposed the same catalytic cycle for both the dark and light reaction, as shown in Scheme 19B for the formation of the product.^64^ Initially, the aminyl radical **54** undergoes SET with the xanthate anion **55**, to obtain the amide anion **59** and xanthate radical **57**. The isolation of Ph_3_P = S (over 95% yield) showed that the phosphoranyl radical **58** was formed in a reaction between P^t^Bu_3_ and the xanthate radical in the next step. The phosphoranyl radical undergoes β-scission, yielding the phosphine sulfide and an alkyl radical. In the last step of the catalytic cycle, the alkyl radical reacts with the pyridinium salt, forming the product and regenerating the aminyl radical **54**. This example of chemical synthesis illustrates that high conversions of the starting material can be obtained if one of the radicals is reactive, even if the equilibrium does not favor the radical state.

## 5 Single-Electron Transfer Facilitated by Lewis Acid Coordination

**Scheme 20:**
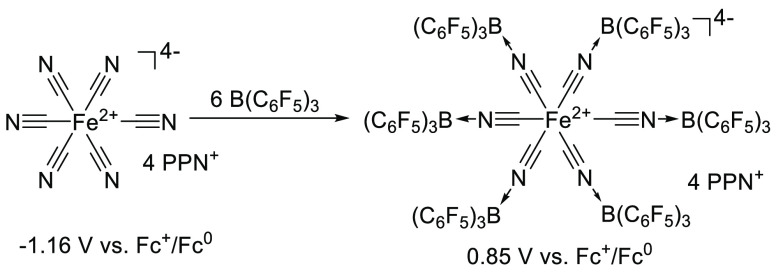
Coordination of B(C_6_F_5_)_3_ as a Lewis Acid to Ferrocyanide Leads to an Increased Reduction Potential and Thus a Higher Electron Affinity PPN^+^ = bis(triphenylphosphine)iminium cation [Ph_3_P = N=PPh_3_]^+^.

As shown in the previous sections, the electron affinity of the Lewis acid and ionization energy of the Lewis base are of fundamental importance for the possibility of SET events. A possible synthetic strategy to widen the range of electron acceptor affinities is the coordination of Lewis acids. An example is the reduction potential of ferrocyanide as reported by the group of Gray et al.^65^ The authors showed that the reduction potential could be altered by as much as 2 V by the coordination of a different number of boranes to the metal (Scheme 20). More examples are abound in organometallic chemistry.^66,67^ Herein, we will present several examples in which the electron affinity of an organic substrate is increased by the coordination of a Lewis acid.

### 5.1 Observations of Single-Electron Transfer Induced by Lewis Acid Coordination

**Scheme 21:**
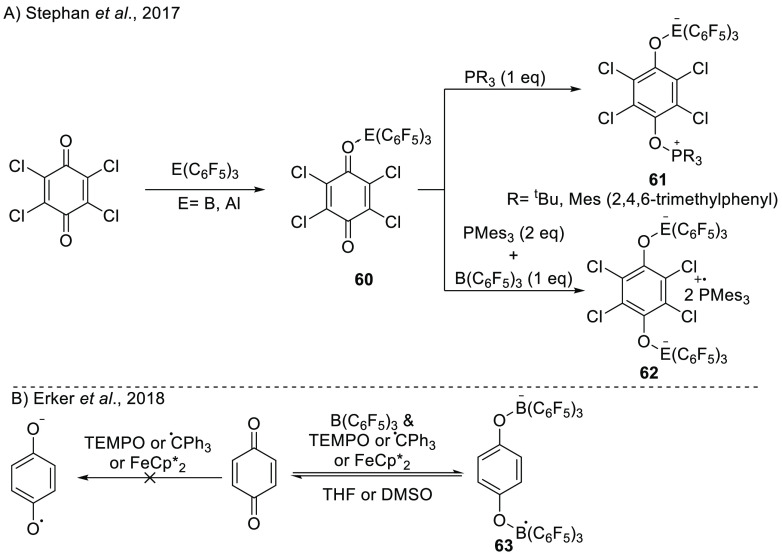
Reduction of Quinones Facilitated by the Coordination of Lewis Acids Using (A) Phosphines as the Electron Donor and (B) TEMPO, Trityl Radical, or Ferrocene as the Electron Donor E = B or Al, R = ^t^Bu or Mes (2,4,6-trimethylphenyl).

Stephan and co-workers found that the FLP systems consisting of B(C_6_F_5_)_3_ or Al(C_6_F_5_)_3_ as a Lewis acid and P^t^Bu_3_ or PMes_3_ as a Lewis base can readily reduce tetrachloro-*p*-benzoquinone (Scheme 21A).^33^ Using equimolar equivalents of P^t^Bu_3_ in toluene at −78 °C, the adduct **60** was found as the product, in which the phosphine forms a covalent bond with the quinone. On the other hand, when PMes_3_ was added in excess, the dianionic *bis*-B(C_6_F_5_)_3_/Al(C_6_F_5_)_3_ adduct **62** resulted, with 2 equiv of the phosphine radical cation as the counterion that was characterized by an absorption at 573 nm in the UV–vis spectrum.

In 2020, Slootweg et al. set out to explore the mechanistic details of these reductions.^17^ They found that both the quinone (EA = 4.45 eV, SCRF/ωB97X-D/6-311+G(d,p) in chlorobenzene) and B(C_6_F_5_)_3_ (EA = 3.31 eV) are clearly not strong enough to oxidize PMes_3_ (5.25 eV) thermally, yet they observed that the reaction proceeds in the dark (Δ*E* = 0.80 eV for *p*-benzoquinone and Δ*E* = 1.94 eV for B(C_6_F_5_)_3_). Encouraged by the report of the groups of Nocera, Jacobsen, and co-workers on the increased electron affinity of quinones by the formation of hydrogen bonds,^68^ Slootweg et al. calculated the electron affinity of the quinone-B(C_6_F_5_)_3_ adduct **60** (Scheme 21A),^17^ which increased by 1.12 to 5.57 eV compared to that of the parent quinone. This electron affinity is now large enough to induce a thermal SET using either P^t^Bu_3_ or PMes_3_ as the Lewis base. Furthermore, the calculations showed that the coordination of a second equivalent of B(C_6_F_5_)_3_ enhances the electron-accepting ability even further, such that a second reduction event can occur with both PMes_3_ and P^t^Bu_3_. In the case of P^t^Bu_3_, the second reduction is prevented by the barrierless formation of the **61** adduct from the semiquinone fragment.

Slootweg et al. also performed spectroscopic studies on the deep purple reaction mixture of PMes_3_/B(C_6_F_5_)_3_ (color comes from EDA complex [PMes_3_, B(C_6_F_5_)_3_]) with 0.5 equiv of tetrachloro-1,4-benzoquinone (TCQ) in the dark.^17^ For this reaction, Stephan et al. had previously reported the presence of the PMes_3_ radical cation.^33^ Slootweg et al. confirmed this by the observation of a doublet signal in the EPR spectrum (*g*_iso_ = 2.0050; *a*_iso_= 23.9 mT) and a triplet multiplet pattern assigned to the formation of the (PMes_3_)_2_^•+^ (*g*_iso_ = 2.0060; *a*_iso_= 16.6 mT); Figure 4). In addition, an undefined featureless signal (*g*_iso_ = 2.0058) was observed, which was assigned to the TCQ centered radical anion, TCQ–B(C_6_F_5_)_3_^•–^. These experimental observations were in-line with the computational result that the SET is preceded by the coordination of B(C_6_F_5_)_3_ to the quinone.

Erker et al. showed the reduction of *p*-benzoquinone in the presence of B(C_6_F_5_)_3_ (Scheme 21B).^69^ By employing TEMPO and the trityl radical or decamethylferrocene (FeCp*_2_) as electron donors, they obtained the radical anion *bis*-B(C_6_F_5_)_3_ adduct **63** (Scheme 21B). Using 2 equiv of the trityl radical, the quinone could be readily reduced to the dianion. However, in the absence of the Lewis acid, none of the electron donors could reduce the quinone moiety. The authors also employed anthraquinone, phenanthrenequinone, and acenaphthenequinone, which all showed similar reactivity to *p*-benzoquinone, although TEMPO was not always a potent enough reductant for these quinones.

Regarding the mechanism for the formation of **63**, the authors found that the reduction of the quinone was reversible, as the addition of THF or DMSO resulted in the backward reaction toward the formation of the quinone.^69^ The addition of these coordinating solvents led to the decoordination of B(C_6_F_5_)_3_ from the quinone and the formation of a Lewis adduct between B(C_6_F_5_)_3_ and THF or DMSO. Consequently, the quinone became less electron accepting and thus induced a BET to yield the quinone.

**Scheme 22:**
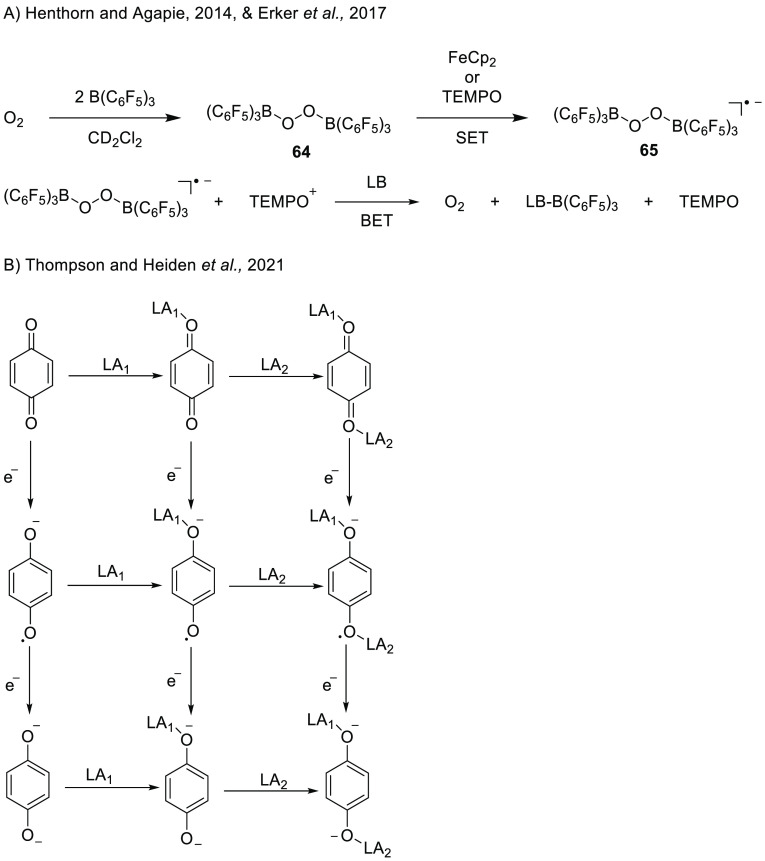
(A) Reduction of Dioxygen Using B(C_6_F_5_)_3_ to Bis(borane)peroxide/Superoxide with Either Ferrocene or TEMPO and BET upon the Addition of THF or DMSO (LB); (B) Pictorial View of the Oxidation States of Quinone and the Lewis Acid Adducts of Each of the Quinone Species LA_1_ = first Lewis acid and LA_2_ = second Lewis acid.

The reduction of dioxygen has been reported by Henthorn and Agapie^70^ and Erker’s group.^71^ First, Henthorn and Agapie showed that even though ferrocene (FeCp_2_) is inert to oxygen, in the presence of 2 equiv of B(C_6_F_5_)_3_ in DCM-*d*_2_, oxygen (1 atm) can be reduced to obtain [(F_5_C_6_)_3_B–O_2_–B(C_6_F_5_)_3_]^2–^ (**64**) in several hours (Scheme 22A). The nature of the product was confirmed by X-ray diffraction, and the control reaction of ferrocene and B(C_6_F_5_)_3_ showed no oxidation of ferrocene, indicating the requirement of both oxygen and B(C_6_F_5_)_3_. In light of the discussed literature, we support the suggestion of the authors that B(C_6_F_5_)_3_ facilities the reduction of oxygen, presumably via coordination in a first step to obtain a strong enough electron donor to be reduced by ferrocene. This is in accordance with the mismatched reduction potentials of ferrocene and O_2_^–^/O_2_ (−1.18 V vs Fc/Fc^+^ in DMSO).^72^

In the subsequent study of Erker et al., the reduction of oxygen (1.5 bar) in DCM-*d*_2_ is achieved using TEMPO as an oxidant in the presence of 2 equiv of B(C_6_F_5_)_3_.^71^ Instead of obtaining the dianion product, TEMPO can achieve only a single reduction toward the [(F_5_C_6_)_3_B–O_2_–B(C_6_F_5_)_3_]^•–^ radical anion (**65)**. This radical was characterized by a seven-line signal at *g*_iso_ = 2.01101 due to hyperfine coupling of both borane nuclei (*a* (^11^B)_iso_ = 0.323 mT). Trapping the B(C_6_F_5_)_3_ with THF or DMSO leads to BET, as observed by the formation of the TEMPO radical and liberation of presumably oxygen gas. As further proof of the feasibility of SET from TEMPO toward the (F_5_C_6_)_3_B–O_2_–B(C_6_F_5_)_3_ adduct, the authors recorded the redox potential of the [(F_5_C_6_)_3_B–O_2_–B(C_6_F_5_)_3_]^−^/[(F_5_C_6_)_3_B–O_2_–B(C_6_F_5_)_3_]^2–^ couple to be +0.22 V vs Fc/Fc^+^ in DCM, which is similar to the TEMPO/TEMPO^+^ redox potential (+0.24 V vs Fc/Fc^+^ in DCM).

Thompson and Heiden also studied the influence of Lewis acids on the reduction of quinones (Lewis acid coordination is shown in Scheme 22B).^73^ Through detailed DFT calculations (SMD/M06-2*X*/6-311++G(d,p) in acetonitrile), they determined that the reduction potential of quinones can be increased by 0.5–1.5 V upon the coordination of a Lewis acid, dependent on the strength of the Lewis acid. For example, the coordination of B(C_6_F_5_)_3_ increased the computed first redox potential by 1.14 V. A further increase of the reduction potential by 0.7–1.6 V was possible by the coordination of a second Lewis acid. In the case of the coordination of 2 equiv of B(C_6_F_5_)_3_, the computed redox potential is 1.16 V compared to −0.96 V for the parent quinone. These results indicate that the electron affinity of quinones can be tuned over a wide range by the addition of Lewis acids, thus facilitating SET processes.

**Scheme 23:**
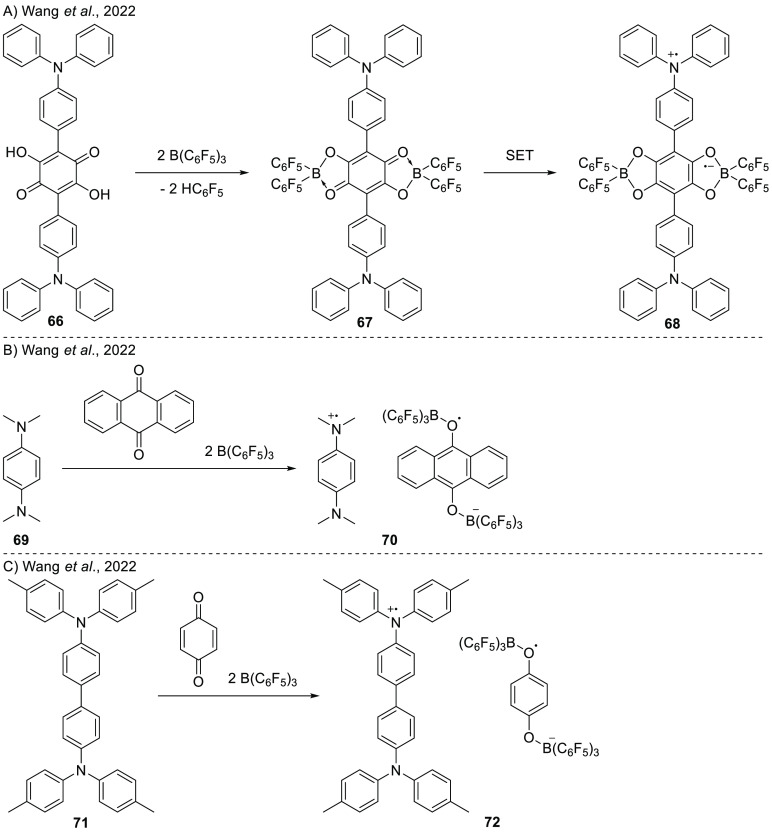
Quinone-Based RIPs Consisting of an Amine-Centered Radical Cation and Boron-Based Radical Anion: (A) D–A–D Molecule by Connecting Two Triarylborane Donors to 1,4-Dihydroxy-benzoquinone; (B) Formation of Structurally Stable RIP (**70**) Using Amine (**69**) and B(C_6_F_5_)_3_ Incorporating Benzoquinone; (C) Formation of RIPs Introducing Benzoquinones as a Link in FLP Reactions via SET Process In all cases, the quinone is made more electron accepting by the coordination of a borane as Lewis acid, thereby facilitating SET processes.

Interested in obtaining an intermolecular SET induced by Lewis acid coordination, Wang’s group investigated the combination of amines as electron donors for borane-activated quinones.^74^ Coordination of the borane to the amine quinone **66** yields the donor–acceptor **67** (Scheme 23A). EPR spectroscopy measurements on a powder sample of **67** revealed Δ*m*_s_ = ±2 half-field absorption features, characteristic of spin-triplet species. The spectrum was characterized by *g*_iso_ = 2.00427, and the zero-field parameters *D* = 14.03 MHz, *E*/*D* = 0.36. The presence of these signals in the EPR spectrum confirmed the presence of a diradical. As **66** in the absence of the Lewis acid was not EPR-active, this clearly demonstrated that the coordination of the borane activates the quinone such that the SET can occur. This was also proven by DFT calculations, which showed for a model substrate an increased EA of −3.71 eV, in comparison to −1.11 eV before borane coordination at the BP86/6-31G(d) level of theory.

**Figure 10:**
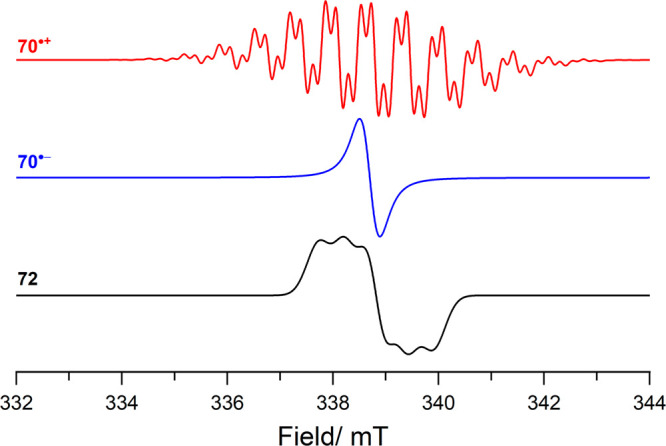
CW X-band EPR spectrum of the individual radical components of **70** (i.e., amine-radical cation **70**^**•+**^ and quinone radical anion **70**^**•–**^), and the radical ion pair **72**; simulated using values listed in Table 1.

In a follow-up study, Wang et al. aimed to demonstrate intermolecular SET events between amines and borane-activated quinones.^75^ Upon mixing amine **69** and 9,10-anthraquinone (Scheme 23B) with 2 equiv of B(C_6_F_5_)_3_ in DCM, the authors obtained a dark blue solution from which they could obtain deep purple crystals. UV–vis spectroscopy of **70** in DCM showed two absorption bands at 570 and 620 nm, which were assigned to the amine radical cation. The crystal structure revealed elongation of several quinone bonds, in tandem with the shortening of amine bonds upon addition, attributed to electron transfer to obtain the quinone radical anion and amine radical cation. The presence of the radical cation and anion were confirmed by the observation of two signals in the EPR spectra (Figure 10). The quinone radical anion gave a featureless signal at *g*_iso_ = 2.0040, while the amine radical cation was observed by a multiplet signal centered at *g*_*i*so_ = 2.0034 with hyperfine splitting originating from coupling to proton and nitrogen nuclei [*a*_iso_(^1^H) = 0.195 and 0.655 mT, and *a*_iso_ (^14^N) = 0.700 mT]. Half-field signals were observed for solid samples (both at room temperature and 88 K), indicating magnetic exchange couplings to be present. SQUID measurements confirmed the relatively strong antiferromagnetic exchange coupling.

In contrast, for the combination of amine **71** and *p*-benzoquinone with 2 equiv of B(C_6_F_5_)_3_, the SQUID measurements showed only weak antiferromagnetic interaction (Scheme 23C).^75^ The authors explained this by the large distance between the cation and anion in the crystal structure. Another difference observed between **69** and **72** was observed in the EPR spectrum. Unlike **69**, for **72** only the radical cation could be observed with a characteristic nitrogen hyperfine coupling [*g*_iso_ = 2.0033, *a*_iso_(^14^N) = 0.45 mT] (Figure 10). The presence of the amine radical cation was further confirmed by UV–vis spectroscopy of a DCM solution of **72**, in which a broad absorption band around 1500 nm was observed that was earlier already ascribed to the amine radical cation using spectroelectrochemistry.^76^ The authors suggest that the lack of observation of the quinone radical anion of **72** was due to a low coupling constant or weak signal intensity.^75^ These results make us on the other hand speculate whether a second reduction of the quinone occurred, yielding a quinone dianion that also could explain the absence of a signal in the EPR spectrum.

**Scheme 24:**
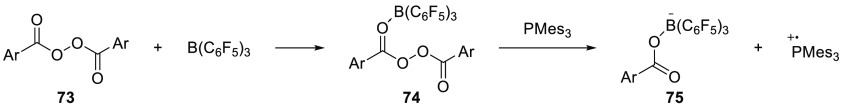
Homolytic Cleavage of the O–O Bond of Benzoyl Peroxides by PMes_3_ in the Presence of B(C_6_F_5_)_3_ Ar = Ph, *p*-MePh, or *p*-BrPh.

Stephan et al. showed the cleavage of the O–O bond of benzoyl peroxides **73** using the FLP system PMes_3_/B(C_6_F_5_)_3_ (Scheme 24).^77^ Previously, the authors observed that B(C_6_F_5_)_3_ coordinates readily to peroxides.^78^ Using Cp*_2_Fe as reductant, it was possible to cleave the O–O bonds of these peroxides. Upon employment of PMes_3_/B(C_6_F_5_)_3_ in combination with 0.5 equiv of (ArCOO)_2_ in DCM, immediately an extremely deep purple solution was obtained containing the salt [PMes_3_]^•+^[RCOOB(C_6_F_5_)_3_]^−^ (R = Ph, *p*-BrC_6_H_5_, *p*-CH_3_C_6_H_5_). The characteristic doublet signal in the EPR spectrum and the absorption maximum at 572 nm further confirmed [PMes_3_]^•+^ formation. The authors proposed the mechanism to start with the coordination of B(C_6_F_5_)_3_ to the ketone moiety of benzoyl peroxide **73**, forming adduct **74**.^77^ Next, the reduction occurred using PMes_3_ as the electron donor. We expect that the increase of the electron affinity of the benzoyl peroxide due to B(C_6_F_5_)_3_ coordination is required to enable the use of PMes_3_ as a one-electron donor.

**Scheme 25:**
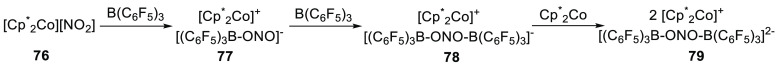
Reduction of Nitrite Anion Using Lewis Acid to Produce Capped Nitrite Mono- and Dianions

Recently, Warren et al. presented the reduction of the nitrite anion using strong Lewis acids, such as B(C_6_F_5_)_3_, without breaking any N–O bonds (Scheme 25). The unsymmetrically capped [Cp*_2_Co][(C_6_F_5_)_3_B–ONO] (**77**) was produced by adding 1 equiv of B(C_6_F_5_)_3_ and [Cp*_2_Co][NO_2_] (**76**) in fluorobenzene and showed the B(C_6_F_5_)_3_ bound to one of the O-atoms of nitrite with distinctly different N–O distances of 1.337(10) Å (for capped O atom) and 1.200(10) Å (free O atom), respectively.^79^ Addition of a second equivalent of B(C_6_F_5_)_3_ in fluorobenzene forms the doubly activated nitrite anion [Cp*_2_Co][(C_6_F_5_)_3_B–ONO–B(C_6_F_5_)_3_] (**78**) with symmetric NO distances (1.261(2), 1.225(2)Å), indicating the coordination of the Lewis acid to both O atoms. No reduction wave was observed either for nitrite (**76**) or monocapped nitrite anion (**77**) in CV using [PPN] [BAr^F^_4_] as the electrolyte in fluorobenzene, whereas the free nitrite is proton-dependent, having a range from +0.98 V and −0.48 V vs NHE (normal hydrogen electrode) at pH 0.0 and 14.0, respectively. A quasi-reversible wave was observed for **78**, centered at −0.74 V versus NHE, corresponding to the [(C_6_F_5_)_3_B–ONO–B(C_6_F_5_)_3_]^−^/[(C_6_F_5_)_3_B– ONO–B(C_6_F_5_)_3_]^2–^ couple. Further chemical reduction of **78** with one more equivalent of Cp*_2_Co in fluorobenzene led to an immediate color change from yellow to gray with the production of borane-capped nitrite radical dianion **79**. Although both the capped mono- and dianions **78** and **79** displayed comparable structure features, IR spectroscopy exhibited a distinct lower-energy N–O stretching frequency for dianion **79** (1.010 cm^–1^), indicating the weakening of the N–O bonds of nitrite upon one-electron reduction, in comparison with monoanion **78** (1.265 cm^–1^). The EPR spectrum for **79** attained the expected isotropic three-line pattern rising from ^14^N hyperfine coupling, with an isotropic hyperfine coupling of 1.57 mT at room temperature. This study showed that besides influencing the redox potential of electron acceptors, also the stability can be influenced at the same time.

### 5.2 Influence of Lewis Acid Coordination on the Photoinduced SET

Interested in fluorescence properties of organic donor–acceptor diads, Abe’s group studied the influence of B(C_6_F_5_)_3_ coordination.^80^ The commercial fluorescence dye **80** has an absorption band in DCM at 422 nm (2.94 eV) for the photoinduced SET from the coumarin core toward the pyridyl group. The fluorescence emission after excitation of the absorption band is at 481 nm (2.58 eV). Upon coordination of B(C_6_F_5_)_3_ as illustrated in Scheme 24, the dye **81** is obtained where the B(C_6_F_5_)_3_ is coordinated to the pyridine acceptor unit of **80**. The absorption of **81** is shifted to 452 nm (2.74 eV) and the fluorescence to 523 nm (2.37 eV). These red shifts are also observed in TD-DFT calculations (B3LYP/6-31G(d) level of theory) where the absorption changes from 373 nm (*f*_osc_ = 0.7108) for **80** to 413 nm (*f*_osc_ = 0.4952) upon B(C_6_F_5_)_3_ coordination.

The authors attributed the observed change in absorption to a smaller HOMO–LUMO gap of **81** (3.30 eV) compared to the 3.57 eV for the parent diad **80** (computed at the B3LYP/6-31G(d) level of theory). The change of orbital energies is also reflected in the change of redox potentials upon the coordination of B(C_6_F_5_)_3_. The oxidation of **80** and **81** showed a small change and are both around 0.5 V vs Fc^+^/Fc in MeCN, while the reduction in DCM went from about −2.3 V vs Fc^+^/Fc to −2.0 V vs Fc^+^/Fc.

**Scheme 26:**
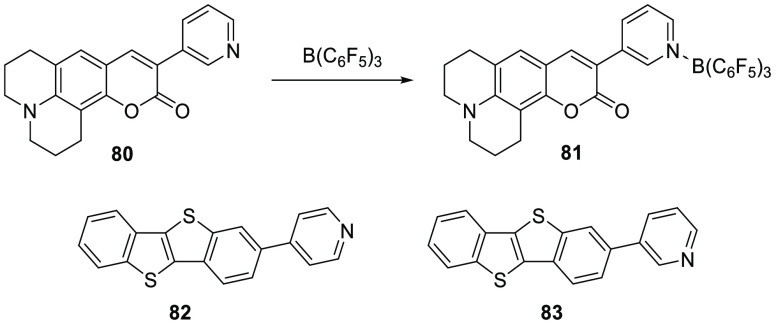
Abe’s Group^80^ Studied Donor–Acceptor Dyes and the Influence of Coordination of B(C_6_F_5_)_3_ at the Acceptor Side

These results demonstrate that the coordination of a Lewis acid to the electron acceptor changes the required wavelength in the case of a photoinduced SET. Besides being reflected in orbital energies and redox potentials, also the fluorescence, likely from BET, moves toward a longer wavelength upon coordination of a Lewis acid. Besides dye **80**, the authors observed similar results for the coordination of B(C_6_F_5_)_3_ to the dyes **82** and **83** (Scheme 26).

### 5.3 Lewis-Acid Catalyzed Single-Electron Transfer: Utilization in Synthesis

**Figure 11:**
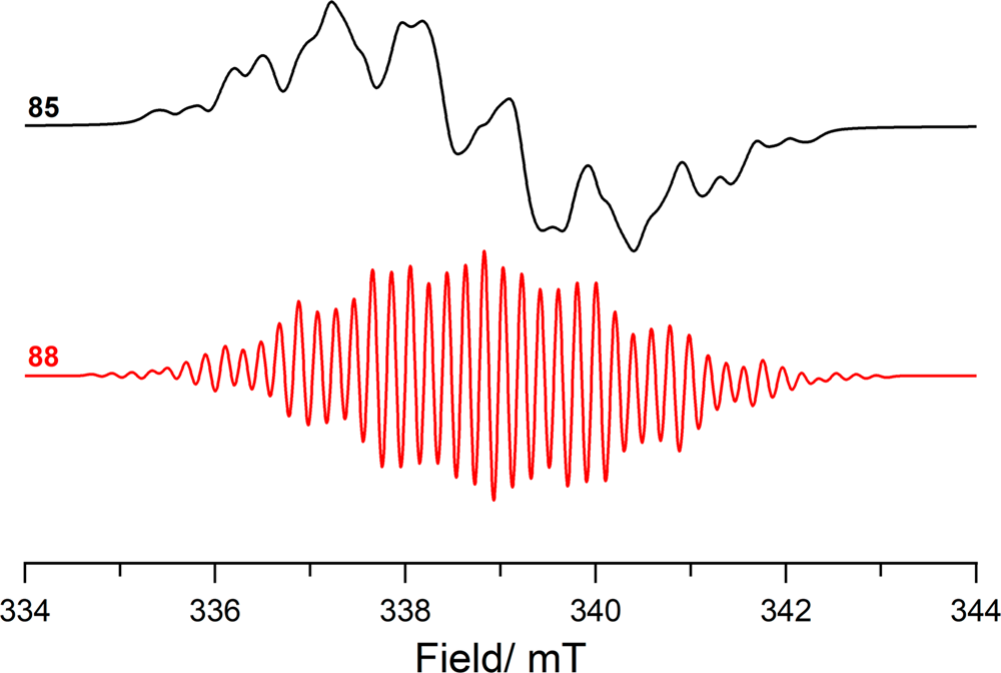
CW X-band EPR spectra of amine radical cations **85** and **88**; simulated using data listed in Table 1.

**Scheme 27:**
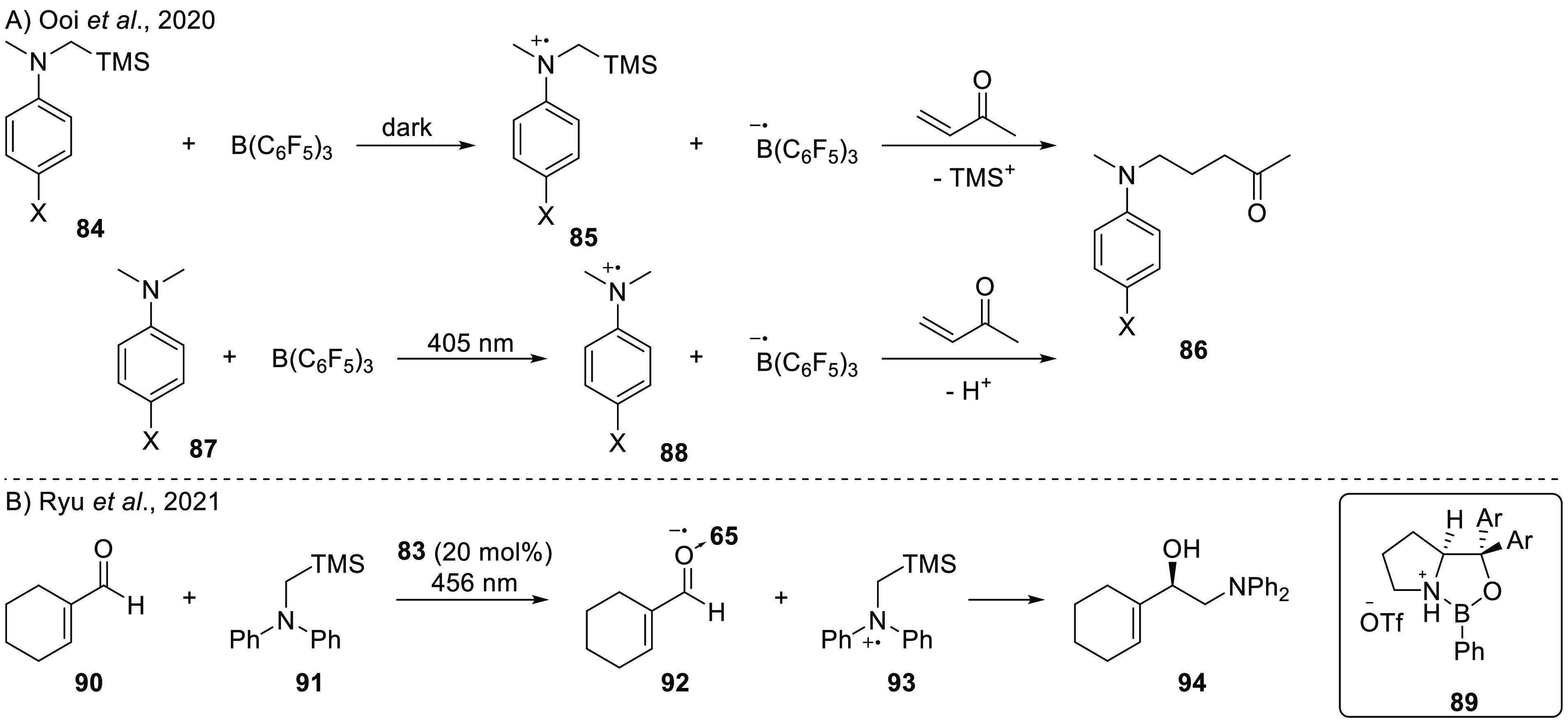
Synthetic Applications of Using SET Facilitated by the Coordination of a Lewis Acid to the Electron Acceptor: (A) Formation of C–C Bonds between Amines and a Vinyl Ketone via the RIP; (B) Formation of an Enantioselective Addition Product via SET Induced by the Coordination of a Chiral Lewis Acid Ar = 2,3-dimethylphenyl, X = Me or Br.

Ooi’s group exploited the use of SET for the formation of C–C bonds between amines and vinyl ketones, catalyzed by B(C_6_F_5_)_3_.^81^ The authors evidenced that thermal SET occurs with B(C_6_F_5_)_3_ and the TMS amine **84** as the amine radical cation **85** could be observed by EPR spectroscopy both in the presence and in the absence of light (characterized by *g*_iso_ = 2.0033 and hyperfine coupling originating from the amine nitrogen and ring protons Figure 11 and Table 1). Upon addition of methyl vinyl ketone, the formation of the addition product **86** was observed (Scheme 27A). The authors purported that this formed via the loss of the TMS cation before the addition of the ketone. Employing the less electron-rich amine **87** (oxidation potential 0.50 V vs Fc/Fc^+^, compared to 0.23 V vs Fc/Fc^+^ for **84**, R = Br in both cases), the authors found that light (405 nm) was required for a reversible SET to occur, as the radical **88** was observed only by EPR upon irradiation with 405 nm (Figure 11 and Table 1). TD-DFT calculations for the EDA complex [**87**, B(C_6_F_5_)_3_] predicted an absorption band at 455 nm (2.74 eV), which allows a photoinduced SET from the amine to B(C_6_F_5_)_3_. The addition of methyl vinyl ketone led to the same coupling product **86**, as was obtained by using **84** as the amine. As in this case irradiation (405 nm) is required, the authors conclude the SET is a step in the mechanism for the formation of product **86**. Later, Slootweg et al. showed that the SET can be facilitated by the coordination of B(C_6_F_5_)_3_ to the oxygen of the ketone. This results in an increased electron affinity of the ketone from −1.43 eV to −2.73 eV (SCRF/ωB97X-D/6-311+G(d,p), in dichloroethane).^17^ This reduces the energy required for SET from the amine **87** toward the ketone from 3.68 to 2.38 eV, making a photoinduced SET feasible during the reaction.

Ryu et al. used the coordination of a chiral Lewis acid **89** to the ketone to obtain an enantioselective addition product (Scheme 27B).^82^ The Lewis acid first coordinates to the aldehyde **90**, which is subsequently able to form an EDA complex with amine **91** (Scheme 27B). The authors proved this using UV–vis spectroscopy, which showed a new absorption peak (a broad peak around 430 nm) in the presence of all three components. Irradiation of this band with 456 nm light promoted photoinduced SET in EDA complex [**91**, **89**] and yielded the RIP consisting of the amine radical cation **93** and the radical anion **92**. The amine radical cation **93** subsequently eliminates the TMS cation, yielding an α-alkyl radical, which couples with the radical anion **92** to obtain the product **94** in good yield (up to 94%) (Scheme 27B). The obtained enantioselectivity (up to 95% ee) further proves that Lewis acid coordination to the aldehyde is required to induce SET.

## 6 Conclusion and Outlook

Since the first report on the simultaneous activation of hydrogen by a Lewis acid and Lewis base, a rise of interest in the chemistry of FLP systems has developed, rejuvenating the field of main group chemistry and catalysis. At first, the reactivity with H_2_ was solely explained via two-electron-transfer pathways, where simultaneous donation and accepting of electron density in the encounter complex occurs. More recently, reports of radicals in FLP chemistry have been appearing. Here, we set out to review the different mechanisms of SET between Lewis base and acid to understand the underlying fundamental processes of radical formation.

One possible way for a SET to occur is photoinduced. In these cases, the absorption of light by the EDA or encounter complex leads to the formation of the radical pair. Light energy is required as the radical state is significantly higher in energy than the ground state electron donor–acceptor complex, which cannot be reached with common thermal means of heating the reaction mixture. Visible-light energy can be used to accomplish a single-electron shift to form transient radical pairs, which are 1.5–3.1 eV higher in energy than the ground state. If the radical state becomes more stable, the SET could occur thermally. For radical states not more than 0.4 eV higher in energy than the ground state, the radicals can potentially be observed in the reaction mixture with EPR spectroscopy at ambient temperature. Larger energy differences will lead to such small quantities of the radical state following the Boltzmann distribution, which makes their detection with, for example, EPR spectroscopy impossible. Yet, at low temperatures (e.g., 30 K) and upon direct irradiation of the sample in the EPR spectrometer, transient high-energy radical ion pairs like PMes_3_^•+^/B(C_6_F_5_)_3_^•–^ have been detected.

Indeed, there are cases where one of the radicals formed following SET is thermally labile and decomposes during the reaction under ambient temperatures, making it only possible to observe the persistent radicals. A consequence of this decomposition is that the equilibrium between the ground state EDA complex and the radical pair shifts toward the radical state. As such, it could be possible to observe radicals even if the radical pairs are too high in energy to be typically observed. In particular, in the case of photoinduced SET, the partial decomposition of the radical pair prevents BET, leading to a buildup of radical concentration. The formation of radicals by SET and subsequent observation can also be facilitated by Lewis acid coordination to the substrate, which acts to increase the electron affinity of the substrate, making the SET event more feasible.

We hope this review leads to more insights and understanding of the SET process in FLP chemistry and related donor–acceptor systems in main group chemistry, which can aid the design of new chemical conversions previously unknown in the two-electron paradigm. During correction of the proofs, the article of Lin et al. was published that highlighted the potential of in-situ generated radical pairs for the regioselective aliphatic C−H functionalization.^85^ More understanding of the fundamental radical steps should help to stir the development of radical mechanisms, ultimately also leading to more selective pathways or higher reaction rates as typical single-electron-transfer steps and radical mechanisms have low energy barriers. It is especially noteworthy that employing irradiation can potentially lead to increased reaction rates through photoinduced SET events. The occurrence of SET in FLP chemistry could also lead to the activation of thus far unreactive substrates or lead to new synthesis pathways.

## References

[ref1] Brown H. C.; Schlesinger H. I.; Cardon S. Z. Studies in Stereochemistry. I. Steric Strains as a Factor in the Relative Stability of Some Coördination Compounds of Boron. J. Am. Chem. Soc. 1942, 64, 325–329. 10.1021/ja01254a031.

[ref2] Welch G. C.; Juan R. R. S.; Masuda J. D.; Stephan D. W. Reversible, Metal-Free Hydrogen Activation. Science 2006, 314, 1124–1126. 10.1126/science.1134230. 17110572

[ref3] McCahill J. S. J.; Welch G. C.; Stephan D. W. Reactivity of “Frustrated Lewis Pairs”: Three-Component Reactions of Phosphines, a Borane, and Olefins. Angew. Chem., Int. Ed. 2007, 46, 4968–4971. 10.1002/anie.200701215. 17526043

[ref4] Sarkar P.; Das S.; Pati S. K. Recent Advances in Group 14 and 15 Lewis Acids for Frustrated Lewis Pair Chemistry. Chem. Asian. J. 2022, 17, e202200148 10.1002/asia.202200148. 35320614

[ref5] Pal R.; Ghara M.; Chattaraj P. K. Activation of Small Molecules and Hydrogenation of CO_2_ Catalyzed by Frustrated Lewis Pairs. Catalysts 2022, 12, 201 10.3390/catal12020201.

[ref6] Tan X.; Wang H. Frustrated Lewis Pair Catalysis: It Takes Two to Make a Thing Go Right. Chin. J. Chem. 2021, 39, 1344–1352. 10.1002/cjoc.202000570.

[ref7] Stephan D. W.; Erker G. Frustrated Lewis Pair Chemistry of Carbon, Nitrogen and Sulfur Oxides. Chem. Sci. 2014, 5, 2625–2641. 10.1039/C4SC00395K.

[ref8] Paradies J. Mechanisms in Frustrated Lewis Pair-Catalyzed Reactions. Eur. J. Org. Chem. 2019, 2019, 283–294. 10.1002/ejoc.201800944.

[ref9] Liu L.; Lukose B.; Jaque P.; Ensing B. Reaction Mechanism of Hydrogen Activation by Frustrated Lewis Pairs. Green Energy & Environment 2019, 4, 20–28. 10.1016/j.gee.2018.06.001.

[ref10] Rokob T. A.; Hamza A.; Stirling A.; Soós T.; Pápai I. Turning Frustration into Bond Activation: A Theoretical Mechanistic Study on Heterolytic Hydrogen Splitting by Frustrated Lewis Pairs. Angew. Chem., Int. Ed. 2008, 47, 2435–2438. 10.1002/anie.200705586. 18288665

[ref11] Jupp A. R. Evidence for the Encounter Complex in Frustrated Lewis Pair Chemistry. Dalton Trans. 2022, 51, 10681–10689. 10.1039/D2DT00655C. 35412552

[ref12] Piers W. E.; Marwitz A. J. V.; Mercier L. G. Mechanistic Aspects of Bond Activation with Perfluoroarylboranes. Inorg. Chem. 2011, 50, 12252–12262. 10.1021/ic2006474. 21612200

[ref13] Habraken E. R. M.; van Leest N. P.; Hooijschuur P.; de Bruin B.; Ehlers A. W.; Lutz M.; Slootweg J. C. Aryldiazonium Salts as Nitrogen-Based Lewis Acids: Facile Synthesis of Tuneable Azophosphonium Salts. Angew. Chem., Int. Ed. 2018, 57, 11929–11933. 10.1002/anie.201806913. 30051582

[ref14] Lawrence E. J.; Oganesyan V. S.; Wildgoose G. G.; Ashley A. E. Exploring the Fate of the Tris(Pentafluorophenyl)Borane Radical Anion in Weakly Coordinating Solvents. Dalton Trans. 2013, 42, 782–789. 10.1039/C2DT31622F. 23201974

[ref15] Welch G. C.; Stephan D. W. Facile Heterolytic Cleavage of Dihydrogen by Phosphines and Boranes. J. Am. Chem. Soc. 2007, 129, 1880–1881. 10.1021/ja067961j. 17260994

[ref16] Holtrop F.; Jupp A. R.; van Leest N. P.; Paradiz Dominguez M.; Williams R. M.; Brouwer A. M.; de Bruin B.; Ehlers A. W.; Slootweg J. C. Photoinduced and Thermal Single-Electron Transfer to Generate Radicals from Frustrated Lewis Pairs. Chem. Eur. J. 2020, 26, 9005–9011. 10.1002/chem.202001494. 32259331 PMC7496419

[ref17] Holtrop F.; Jupp A. R.; Kooij B. J.; van Leest N. P.; de Bruin B.; Slootweg J. C. Single-Electron Transfer in Frustrated Lewis Pair Chemistry. Angew. Chem., Int. Ed. 2020, 132, 22394–22400. 10.1002/ange.202009717. PMC7756365 32840947

[ref18] Zheng X.; Wang X.; Qiu Y.; Li Y.; Zhou C.; Sui Y.; Li Y.; Ma J.; Wang X. One-Electron Oxidation of an Organic Molecule by B(C_6_F_5_)_3_; Isolation and Structures of Stable Non-Para-Substituted Triarylamine Cation Radical and Bis(Triarylamine) Dication Diradicaloid. J. Am. Chem. Soc. 2013, 135, 14912–14915. 10.1021/ja407318h. 24053534

[ref19] Kwaan R. J.; Harlan C. J.; Norton J. R. Generation and Characterization of the Tris(Pentafluorophenyl)Borane Radical Anion. Organometallics 2001, 20, 3818–3820. 10.1021/om010272q.

[ref20] Merk A.; Großekappenberg H.; Schmidtmann M.; Luecke M.-P.; Lorent C.; Driess M.; Oestreich M.; Klare H. F. T.; Müller T. Single-Electron Transfer Reactions in Frustrated and Conventional Silylium Ion/Phosphane Lewis Pairs. Angew. Chem., Int. Ed. 2018, 57, 15267–15271. 10.1002/anie.201808922. 30178534

[ref21] Murugesan R.; Subramanian S. E.P.R. of Radicals in γ-Irradiated Substituted Phosphines. Mol. Phys. 1979, 38, 1941–1953. 10.1080/00268977900102961.

[ref22] Dasgupta A.; Richards E.; Melen R. L. Frustrated Radical Pairs: Insights from EPR Spectroscopy. Angew. Chem., Int. Ed. 2021, 60, 53–65. 10.1002/anie.202010633. PMC7883636 32931604

[ref23] Holtrop F.; Jupp A.; Slootweg J. C. Radicals in Frustrated Lewis Pair Chemistry. In Frustrated Lewis Pairs; Jupp A. R., Slootweg J. C., Eds.; Molecular Catalysis (MOLCAT); Springer, Cham, 2021; pp 361–385.

[ref24] Liu L. L.; Stephan D. W. Radicals Derived from Lewis Acid/Base Pairs. Chem. Soc. Rev. 2019, 48, 3454–3463. 10.1039/C8CS00940F. 30724924

[ref25] Wass D. F.; Chapman A. M. Frustrated Lewis Pairs Beyond the Main Group: Transition Metal-Containing Systems. In Frustrated Lewis Pairs II: Expanding the Scope; Erker G., Stephan D. W., Eds.; Topics in Current Chemistry; Springer: Berlin, Heidelberg, 2013; pp 261–280. 10.1007/128_2012_395 23468285

[ref26] Cardenas A. J. P.; Culotta B. J.; Warren T. H.; Grimme S.; Stute A.; Fröhlich R.; Kehr G.; Erker G. Capture of NO by a Frustrated Lewis Pair: A New Type of Persistent N-Oxyl Radical. Angew. Chem., Int. Ed. 2011, 50, 7567–7571. 10.1002/anie.201101622. 21726024

[ref27] Sajid M.; Kehr G.; Wiegand T.; Eckert H.; Schwickert C.; Pöttgen R.; Cardenas A. J. P.; Warren T. H.; Fröhlich R.; Daniliuc C. G.; Erker G. Noninteracting, Vicinal Frustrated P/B-Lewis Pair at the Norbornane Framework: Synthesis, Characterization, and Reactions. J. Am. Chem. Soc. 2013, 135, 8882–8895. 10.1021/ja400338e. 23627402

[ref28] Pereira J. C. M.; Sajid M.; Kehr G.; Wright A. M.; Schirmer B.; Qu Z.-W.; Grimme S.; Erker G.; Ford P. C. Reaction of a Bridged Frustrated Lewis Pair with Nitric Oxide: A Kinetics Study. J. Am. Chem. Soc. 2014, 136, 513–519. 10.1021/ja4118335. 24328325

[ref29] Özgün T.; Chen G.-Q.; Daniliuc C. G.; McQuilken A. C.; Warren T. H.; Knitsch R.; Eckert H.; Kehr G.; Erker G. Unsaturated Vicinal Frustrated Lewis Pair Formation by Electrocyclic Ring Closure and Their Reaction with Nitric Oxide. Organometallics 2016, 35, 3667–3680. 10.1021/acs.organomet.6b00627.

[ref30] Liedtke R.; Scheidt F.; Ren J.; Schirmer B.; Cardenas A. J. P.; Daniliuc C. G.; Eckert H.; Warren T. H.; Grimme S.; Kehr G.; Erker G. Frustrated Lewis Pair Modification by 1,1-Carboboration: Disclosure of a Phosphine Oxide Triggered Nitrogen Monoxide Addition to an Intramolecular P/B Frustrated Lewis Pair. J. Am. Chem. Soc. 2014, 136, 9014–9027. 10.1021/ja5028293. 24850528

[ref31] Sajid M.; Stute A.; Cardenas A. J. P.; Culotta B. J.; Hepperle J. A. M.; Warren T. H.; Schirmer B.; Grimme S.; Studer A.; Daniliuc C. G.; Fröhlich R.; Petersen J. L.; Kehr G.; Erker G. *N*, *N* -Addition of Frustrated Lewis Pairs to Nitric Oxide: An Easy Entry to a Unique Family of Aminoxyl Radicals. J. Am. Chem. Soc. 2012, 134, 10156–10168. 10.1021/ja302652a. 22548454

[ref32] Ménard G.; Hatnean J. A.; Cowley H. J.; Lough A. J.; Rawson J. M.; Stephan D. W. C–H Bond Activation by Radical Ion Pairs Derived from R_3_P/Al(C_6_F_5_)_3_ Frustrated Lewis Pairs and N_2_O. J. Am. Chem. Soc. 2013, 135, 6446–6449. 10.1021/ja402964h. 23594345

[ref33] Liu L. L.; Cao L. L.; Shao Y.; Ménard G.; Stephan D. W. A Radical Mechanism for Frustrated Lewis Pair Reactivity. Chem. 2017, 3, 259–267. 10.1016/j.chempr.2017.05.022.

[ref34] Mulliken R. S. Molecular Compounds and Their Spectra. II. J. Am. Chem. Soc. 1952, 74, 811–824. 10.1021/ja01123a067.

[ref35] Rosokha S. V.; Kochi J. K. Fresh Look at Electron-Transfer Mechanisms via the Donor/Acceptor Bindings in the Critical Encounter Complex. Acc. Chem. Res. 2008, 41, 641–653. 10.1021/ar700256a. 18380446

[ref36] Silvi M.; Melchiorre P. Enhancing the Potential of Enantioselective Organocatalysis with Light. Nature 2018, 554, 41–49. 10.1038/nature25175. 29388950

[ref37] Marques L. R.; Ando R. A. Probing the Charge Transfer in a Frustrated Lewis Pair by Resonance Raman Spectroscopy and DFT Calculations. ChemPhysChem 2021, 22, 522–525. 10.1002/cphc.202001024. 33512751

[ref38] Adelizzi B.; Chidchob P.; Tanaka N.; Lamers B. A. G.; Meskers S. C. J.; Ogi S.; Palmans A. R. A.; Yamaguchi S.; Meijer E. W. Long-Lived Charge-Transfer State from B–N Frustrated Lewis Pairs Enchained in Supramolecular Copolymers. J. Am. Chem. Soc. 2020, 142, 16681–16689. 10.1021/jacs.0c06921. 32880167 PMC7530894

[ref39] Chidchob P.; Jansen S. A. H.; Meskers S. C. J.; Weyandt E.; van Leest N. P.; de Bruin B.; Palmans A. R. A.; Vantomme G.; Meijer E. W. Supramolecular Systems Containing B–N Frustrated Lewis Pairs of Tris(Pentafluorophenyl)Borane and Triphenylamine Derivatives. Organic Materials 2021, 03, 174–183. 10.1055/s-0041-1727235.

[ref40] Jin T.; Bolte M.; Lerner H.-W.; Mewes J.-M.; Wagner M. Charge-Transfer Transitions Govern the Reactivity and Photophysics of Vicinally Diphosphanyl-Substituted Diborapentacenes. Chem. Eur. J. 2022, 28, e202202234 10.1002/chem.202202234. 36094675 PMC9826252

[ref41] Yuan W.; Huang J.; Xu X.; Wang L.; Tang X.-Y. B(C_6_F_5_)_3_-Catalyzed Electron Donor–Acceptor Complex-Mediated Aerobic Sulfenylation of Indoles under Visible-Light Conditions. Org. Lett. 2021, 23, 7139–7143. 10.1021/acs.orglett.1c02553. 34449237

[ref42] Ishikawa R.; Iwasawa R.; Takiyama Y.; Yamauchi T.; Iwanaga T.; Takezaki M.; Watanabe M.; Teramoto N.; Shimasaki T.; Shibata M. Synthesis of 1,2-Bis(2-Aryl-1H-Indol-3-Yl)Ethynes via 5-Exo-Digonal Double Cyclization Reactions of 1,4-Bis(2-Isocyanophenyl)Buta-1,3-Diyne with Aryl Grignard Reagents. J. Org. Chem. 2017, 82, 652–663. 10.1021/acs.joc.6b02668. 27982589

[ref43] Li S.; Hu C.; Cui X.; Zhang J.; Liu L. L.; Wu L. Site-Fixed Hydroboration of Terminal and Internal Alkenes Using BX_3_/IPr_2_NEt. Angew. Chem., Int. Ed. 2021, 60, 26238–26245. 10.1002/anie.202111978. 34536251

[ref44] van Dalsen L.; Brown R. E.; Rossi-Ashton J. A.; Procter D. J. Sulfonium Salts as Acceptors in Electron Donor-Acceptor Complexes. Angew. Chem., Int. Ed. 2023, e202303104 10.1002/ange.202303104. PMC10952135 36959098

[ref45] Bednar T. N.; Nagib D. A. Radical Arenes. Nat. Chem. 2023, 15, 3–4. 10.1038/s41557-022-01109-6. 36522580

[ref46] Dewanji A.; van Dalsen L.; Rossi-Ashton J. A.; Gasson E.; Crisenza G. E. M.; Procter D. J. A General Arene C–H Functionalization Strategy via Electron Donor–Acceptor Complex Photoactivation. Nat. Chem. 2023, 15, 43–52. 10.1038/s41557-022-01092-y. 36471045

[ref47] Hamann B. C.; Hartwig J. F. Palladium-Catalyzed Direct α-Arylation of Ketones. Rate Acceleration by Sterically Hindered Chelating Ligands and Reductive Elimination from a Transition Metal Enolate Complex. J. Am. Chem. Soc. 1997, 119, 12382–12383. 10.1021/ja9727880.

[ref48] Escudero-Casao M.; Licini G.; Orlandi M. Enantioselective α-Arylation of Ketones via a Novel Cu(I)–Bis(Phosphine) Dioxide Catalytic System. J. Am. Chem. Soc. 2021, 143, 3289–3294. 10.1021/jacs.0c13236. 33635068 PMC8041290

[ref49] Zhao D.; Xu P.; Ritter T. Palladium-Catalyzed Late-Stage Direct Arene Cyanation. Chem. 2019, 5, 97–107. 10.1016/j.chempr.2018.09.027.

[ref50] McManus J. B.; Nicewicz D. A. Direct C–H Cyanation of Arenes via Organic Photoredox Catalysis. J. Am. Chem. Soc. 2017, 139, 2880–2883. 10.1021/jacs.6b12708. 28177237 PMC5541851

[ref51] Möser J.; Lips K.; Tseytlin M.; Eaton G. R.; Eaton S. S.; Schnegg A. Using Rapid-Scan EPR to Improve the Detection Limit of Quantitative EPR by More than One Order of Magnitude. J. Magn. Reson. 2017, 281, 17–25. 10.1016/j.jmr.2017.04.003. 28500917 PMC5556260

[ref52] Hoffmann K. F.; Battke D.; Golz P.; Rupf S. M.; Malischewski M.; Riedel S. The Tris(Pentafluorophenyl)Methylium Cation: Isolation and Reactivity. Angew. Chem., Int. Ed. 2022, 61, e202203777 10.1002/anie.202203777. PMC9401592 35416383

[ref53] Shaikh A. C.; Veleta J. M.; Moutet J.; Gianetti T. L. Trioxatriangulenium (TOTA^+^) as a Robust Carbon-Based Lewis Acid in Frustrated Lewis Pair Chemistry. Chem. Sci. 2021, 12, 4841–4849. 10.1039/D0SC05893A. 34168760 PMC8179643

[ref54] Schmidlin N. M. C.; Radtke V.; Schmidt A.; Lõkov M.; Leito I.; Böttcher T. Electronic Modification of a Sterically Demanding Anionic Pyridine Ligand. Z. Anorg. Allg. Chem. 2022, 648, e202200136 10.1002/zaac.202200136.

[ref55] Waked A. E.; Ostadsharif Memar R.; Stephan D. W. Nitrogen-Based Lewis Acids Derived from Phosphonium Diazo Cations. Angew. Chem., Int. Ed. 2018, 57, 11934–11938. 10.1002/anie.201804183. 29806886

[ref56] Soltani Y.; Dasgupta A.; Gazis T. A.; Ould D. M. C.; Richards E.; Slater B.; Stefkova K.; Vladimirov V. Y.; Wilkins L. C.; Willcox D.; Melen R. L. Radical Reactivity of Frustrated Lewis Pairs with Diaryl Esters. Cell Rep. Phys. Sci. 2020, 1, 100016 10.1016/j.xcrp.2020.100016.

[ref57] Dasgupta A.; Stefkova K.; Babaahmadi R.; Yates B. F.; Buurma N. J.; Ariafard A.; Richards E.; Melen R. L. Site-Selective Csp^3^–Csp/Csp^3^–Csp^2^ Cross-Coupling Reactions Using Frustrated Lewis Pairs. J. Am. Chem. Soc. 2021, 143, 4451–4464. 10.1021/jacs.1c01622. 33719443 PMC8041292

[ref84] Albrecht P. A.; Rupf S.; Sellin M.; Schlögl J.; Riedel S.; Malischewski M. Increasing the oxidation power of TCNQ by coordination of B(C_6_F_5_)_3_. Chem. Commun. 2022, 58, 4958–4961. 10.1039/D2CC00314G. 35380574

[ref58] Kochi J. K. Inner-Sphere Electron Transfer in Organic Chemistry. Relevance to Electrophilic Aromatic Nitration. Acc. Chem. Res. 1992, 25, 39–47. 10.1021/ar00013a006.

[ref59] Dong Z.; Cramer H. H.; Schmidtmann M.; Paul L. A.; Siewert I.; Müller T. Evidence for a Single Electron Shift in a Lewis Acid–Base Reaction. J. Am. Chem. Soc. 2018, 140, 15419–15424. 10.1021/jacs.8b09214. 30359019

[ref60] De Heer J. The Principle of Le Châtelier and Braun. J. Chem. Educ. 1957, 34, 375 10.1021/ed034p375.

[ref61] Schilter D. Frustration Leads to Radical Behaviour. Nat. Rev. Chem. 2018, 2, 255–255. 10.1038/s41570-018-0047-1.

[ref62] Gynane M. J. S.; Lappert M. F.; Riley P. I.; Rivière P.; Rivière-Baudet M. Triaryl-Silyl, -Germyl, and -Stannyl Radicals. MAr_3_ (M = Si, Ge, or Sn and Ar = 2,4,6-Me_3_C_6_H_2_) and Ge(2,6-Me_2_C_6_H_3_)_3_: Synthesis and ESR Studies. J. Organomet. Chem. 1980, 202, 5–12. 10.1016/S0022-328X(00)81378-9.

[ref63] Dong Z.; Pezzato C.; Sienkiewicz A.; Scopelliti R.; Fadaei-Tirani F.; Severin K. SET Processes in Lewis Acid–Base Reactions: The Tritylation of N-Heterocyclic Carbenes. Chem. Sci. 2020, 11, 7615–7618. 10.1039/D0SC01278E. 34094138 PMC8159480

[ref64] Tan C.-Y.; Kim M.; Park I.; Kim Y.; Hong S. Site-Selective Pyridine C–H Alkylation with Alcohols and Thiols via Single-Electron Transfer of Frustrated Lewis Pairs. Angew. Chem., Int. Ed. 2022, 61, e202213857 10.1002/anie.202213857. 36314414

[ref65] McNicholas B. J.; Grubbs R. H.; Winkler J. R.; Gray H. B.; Despagnet-Ayoub E. Tuning the Formal Potential of Ferrocyanide over a 2.1 V Range. Chem. Sci. 2019, 10, 3623–3626. 10.1039/C8SC04972F. 30996955 PMC6430091

[ref66] Sankaralingam M.; Lee Y.-M.; Nam W.; Fukuzumi S. Amphoteric Reactivity of Metal–Oxygen Complexes in Oxidation Reactions. Coord. Chem. Rev. 2018, 365, 41–59. 10.1016/j.ccr.2018.03.003.

[ref67] Fukuzumi S.; Ohkubo K.; Lee Y.-M.; Nam W. Lewis Acid Coupled Electron Transfer of Metal–Oxygen Intermediates. Chem. Eur. J. 2015, 21, 17548–17559. 10.1002/chem.201502693. 26404482

[ref68] Turek A. K.; Hardee D. J.; Ullman A. M.; Nocera D. G.; Jacobsen E. N. Activation of Electron-Deficient Quinones through Hydrogen-Bond-Donor-Coupled Electron Transfer. Angew. Chem., Int. Ed. 2016, 55, 539–544. 10.1002/anie.201508060. PMC5120606 26612607

[ref69] Tao X.; Daniliuc C. G.; Knitsch R.; Hansen M. R.; Eckert H.; Lübbesmeyer M.; Studer A.; Kehr G.; Erker G. The Special Role of B(C_6_F_5_)_3_ in the Single Electron Reduction of Quinones by Radicals. Chem. Sci. 2018, 9, 8011–8018. 10.1039/C8SC03005G. 30450185 PMC6202917

[ref70] Henthorn J. T.; Agapie T. Dioxygen Reactivity with a Ferrocene–Lewis Acid Pairing: Reduction to a Boron Peroxide in the Presence of Tris(Pentafluorophenyl)Borane. Angew. Chem., Int. Ed. 2014, 126, 13107–13110. 10.1002/ange.201408462. 25250531

[ref71] Tao X.; Daniliuc C. G.; Janka O.; Pöttgen R.; Knitsch R.; Hansen M. R.; Eckert H.; Lübbesmeyer M.; Studer A.; Kehr G.; Erker G. Reduction of Dioxygen by Radical/B(*p*-C_6_F_4_X)_3_ Pairs to Give Isolable Bis(Borane)Superoxide Compounds. Angew. Chem., Int. Ed. 2017, 56, 16641–16644. 10.1002/anie.201709309. 29112325

[ref72] Maricle D. L.; Hodgson W. G. Reducion of Oxygen to Superoxide Anion in Aprotic Solvents. Anal. Chem. 1965, 37, 1562–1565. 10.1021/ac60231a027.

[ref73] Thompson B. L.; Heiden Z. M. Tuning the Reduction Potentials of Benzoquinone through the Coordination to Lewis Acids. Phys. Chem. Chem. Phys. 2021, 23, 9822–9831. 10.1039/D1CP01266E. 33908513

[ref74] Wang J.; Cui H.; Ruan H.; Zhao Y.; Zhao Y.; Zhang L.; Wang X. The Lewis Acid Induced Formation of a Stable Diradical with an Intramolecular Ion Pairing State. J. Am. Chem. Soc. 2022, 144, 7978–7982. 10.1021/jacs.2c02902. 35485969

[ref75] Kong S.; Tang S.; Wang T.; Zhao Y.; Sun Q.; Zhao Y.; Wang X. Stable Radical Ion Pairs Induced by Single Electron Transfer: Frustrated Versus Nonfrustrated. CCS Chemistry 2023, 5, 334–340. 10.31635/ccschem.022.202202306.

[ref76] Low P. J.; Paterson M. A. J.; Goeta A. E.; Yufit D. S.; Howard J. A. K.; Cherryman J. C.; Tackley D. R.; Brown B. The Molecular Structures and Electrochemical Response of “Twisted” Tetra(Aryl)Benzidenes. J. Mater. Chem. 2004, 14, 2516–2523. 10.1039/B404731A.

[ref77] Liu L. L.; Cao L. L.; Zhu D.; Zhou J.; Stephan D. W. Homolytic Cleavage of Peroxide Bonds via a Single Electron Transfer of a Frustrated Lewis Pair. Chem. Commun. 2018, 54, 7431–7434. 10.1039/C8CC03522A. 29781011

[ref78] Liu L. L.; Cao L. L.; Shao Y.; Stephan D. W. Single Electron Delivery to Lewis Pairs: An Avenue to Anions by Small Molecule Activation. J. Am. Chem. Soc. 2017, 139, 10062–10071. 10.1021/jacs.7b05120. 28654746

[ref79] Hosseininasab V.; DiMucci I. M.; Ghosh P.; Bertke J. A.; Chandrasekharan S.; Titus C. J.; Nordlund D.; Freed J. H.; Lancaster K. M.; Warren T. H. Lewis Acid-Assisted Reduction of Nitrite to Nitric and Nitrous Oxides via the Elusive Nitrite Radical Dianion. Nat. Chem. 2022, 14, 1265–1269. 10.1038/s41557-022-01025-9. 36064970 PMC9633411

[ref80] Ikeda T.; Tahara K.; Ishimatsu R.; Ono T.; Cui L.; Maeda M.; Ozawa Y.; Abe M. Lewis-Pairing-Induced Electrochemiluminescence Enhancement from Electron Donor-Acceptor Diads Decorated with Tris(Pentafluorophenyl)Borane as an Electrochemical Protector. Angew. Chem., Int. Ed. 2023, 62, e202301109 10.1002/anie.202304251. 36878874

[ref81] Aramaki Y.; Imaizumi N.; Hotta M.; Kumagai J.; Ooi T. Exploiting Single-Electron Transfer in Lewis Pairs for Catalytic Bond-Forming Reactions. Chem. Sci. 2020, 11, 4305–4311. 10.1039/D0SC01159B. 34122888 PMC8152713

[ref82] Kim J. Y.; Lee Y. S.; Ryu D. H. Ternary Electron Donor–Acceptor Complex Enabled Enantioselective Radical Additions to α, β-Unsaturated Carbonyl Compounds. ACS Catal. 2021, 11, 14811–14818. 10.1021/acscatal.1c04835.

[ref83] Symons M. C. R.; Tordo P.; Wyatt J. The Structure of Diphosphine Radical Cations. J. Organomet. chem. 1993, 443, C29–C32. 10.1016/0022-328X(93)80310-8.

[ref85] Lu Z.; Ju M.; Wang Y.; Meinhardt J. M.; Alvarado J. I. M.; Villemure E.; Terrett J. A.; Lin S. Regioselective aliphatic C–H functionalization using frustrated radical pairs. Nature 2023, 10.1038/s41586-023-06131-3. PMC10530363 37407819

